# Macrophage Phenotype–Dependent Protein Corona Formation Governs Ligand Accessibility and Immune Clearance of Biomimetic Nanoparticles

**DOI:** 10.1002/smll.202514389

**Published:** 2026-02-20

**Authors:** Tianchang He, Lina Zhu, Jiayi Ding, Xiaoyan Fang, Yu Gao, Volker Mailänder, Daniel Crespy, Katharina Landfester, Shuai Jiang

**Affiliations:** ^1^ Key Laboratory of Marine Drugs Chinese Ministry of Education School of Medicine and Pharmacy Ocean University of China Qingdao P. R. China; ^2^ State Key Laboratory of Marine Food Processing and Safety Control Ocean University of China Qingdao P. R. China; ^3^ Laboratory for Marine Drugs and Bioproducts Qingdao Marine Science and Technology Center Qingdao P. R. China; ^4^ Max Planck Institute for Polymer Research Mainz Germany; ^5^ Dermatology Department University Medicine Mainz Mainz Germany; ^6^ Department of Materials Science and Engineering School of Molecular Science and Engineering Vidyasirimedhi Institute of Science and Technology (VISTEC) Rayong Thailand

**Keywords:** biomimetic nanoparticles, complement activation, immune clearance, macrophage phenotype, protein corona

## Abstract

The phenotype of source cells used for membrane coating fundamentally influences the biointerfacing behavior of cell membrane‐coated nanoparticles. Meanwhile, the formation of a protein corona (PC) in biological fluids plays a pivotal role in dictating the in vivo fate of nanoparticles. Yet, how macrophage phenotypes influence PC composition and, in turn, dictate the clearance of biomimetic nanoparticles remains underexplored. Here, we prepared magnetic silica nanoparticles (SMNs) coated with membranes from M0, M1, and M2 macrophages (denoted as M0@SMNs, M1@SMNs, and M2@SMNs) to elucidate phenotype‐dependent PC fingerprints and their impact on immune recognition and clearance. In vivo and in vitro studies demonstrated that M0@SMNs exhibited superior immune evasion, reduced hepatic clearance, and prolonged blood retention. Nano‐flow cytometry revealed that PC formation masked up to ≈40% of surface membrane proteins. Furthermore, proteomics, Western blotting, and ELISA analyses confirmed that M0@SMNs exhibited minimal adsorption of immune opsonins (C3, IgG, IgM) and triggered the lowest complement activation, which account for their attenuated hepatic clearance. Collectively, these findings identify source cell phenotype as a key determinant of PC composition and clearance fate, thereby offering mechanistic guidance for the rational design of biomimetic nanocarriers. Notably, M0 macrophages confer superior systemic circulation relative to M1 and M2 counterparts.

## Graphical abstract

Macrophage phenotype dictates protein corona composition and immune clearance of biomimetic nanoparticles. M0‐derived membranes minimize opsonin adsorption and complement activation, enabling superior immune evasion and prolonged circulation, thereby providing mechanistic insights for designing long‐circulating nanocarriers.

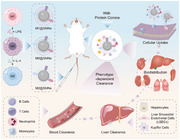

## 1 Introduction

Nanomedicine has attracted significant attention for its ability to enhance drug solubility, prolong systemic circulation, and achieve targeted delivery with reduced off‐target effects [1, 2, 3]. Among various approaches, biomimetic nanoparticles cloaked in natural cell membranes (CM‐NPs) have emerged as a promising platform, offering superior biocompatibility [4], immune evasion [5], extended circulation [6, 7], and disease‐specific targeting [8, 9, 10, 11]. A variety of membrane sources have been explored—including erythrocytes [12, 13], platelets [14], tumor cells [15, 16], and immune cells [17, 18]—each conferring distinct biological properties to the nanoparticles. Notably, macrophage membranes have attracted growing interest due to their inherent inflammation‐homing capacity [19] and low immunogenicity [20, 21]. Moreover, macrophages exhibit remarkable phenotypic plasticity and can polarize into M0 (naïve), M1 (pro‐inflammatory), or M2 (anti‐inflammatory) subtypes, each with unique membrane protein compositions, tissue distribution, and immunological functions [22, 23]. These phenotypic distinctions have inspired the application of macrophage membrane‐coated nanoparticles (M@NPs) in various disease contexts. For instance, M0 membranes prolong circulation and enhance immune evasion [19, 24, 25]. M1 membranes exploit pro‐inflammatory activity for antitumor immunity including metastasis inhibition [26, 27] and checkpoint blockade potentiation [28], while M2 membranes provide anti‐inflammatory and regenerative benefits in ARDS [29], atherosclerosis therapy [30], and targeted imaging [31]. Despite these advances, the implications of macrophage phenotype on nanoparticle–protein interactions remain underexplored. Given their distinct immunological roles, M0, M1, and M2 membranes likely differ in how they interact with serum proteins—particularly immunoglobulins, acute‐phase proteins, and complement components—which in turn may influence nanoparticle clearance and biodistribution. However, these effects have not been systematically investigated.

Upon intravenous injection, nanoparticles are rapidly immersed in complex biological fluids where plasma proteins adsorb onto their surfaces to form a dynamic protein corona (PC) [32, 33, 34, 35]. This corona redefines the nanoparticle's surface identity by masking native membrane ligands, altering biological interactions, and triggering immune clearance *via* the mononuclear phagocyte system [36, 37, 38]. Among the key components of the PC, immune opsonins such as IgG, IgM, and complement factors (e.g., C3) are commonly found, which play central roles in mediating phagocytic uptake and hepatic clearance [39, 40]. While extensive research has addressed PC formation on synthetic nanoparticles, the influence of natural membrane coatings—especially those derived from immunologically active macrophages—on PC composition, membrane ligand shielding, and complement activation remains largely unknown.

**SCHEME 1:**
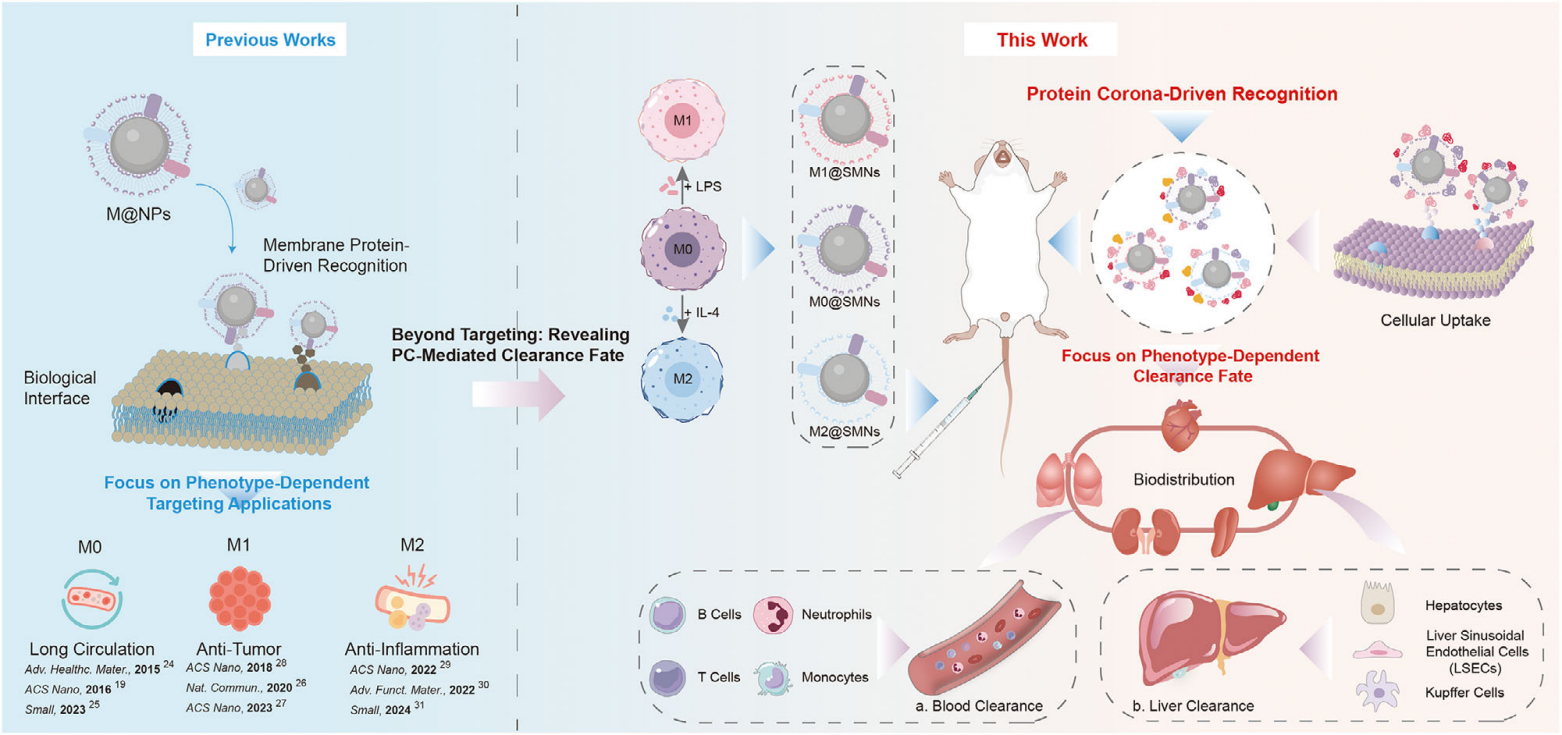
Schematic illustration of phenotype‐dependent protein corona formation on macrophage membrane–coated nanoparticles (M@SMNs) and its impact on systemic clearance. Previous studies primarily focused on the phenotype‐dependent targeting applications of M@NPs, including prolonged circulation by M0‐derived membranes [19, 24, 25], anti‐tumor responses by M1‐derived membranes [26, 27, 28], and anti‐inflammatory effects by M2‐derived membranes [29, 30, 31], while largely overlooking the role of the protein corona. In this study, macrophages were either left untreated (M0) or polarized into M1 and M2 phenotypes *via* LPS or IL‐4 stimulation to generate M0@SMNs, M1@SMNs, and M2@SMNs. Upon intravenous injection, M@SMNs formed distinct phenotype‐specific protein corona in the bloodstream, which in turn modulated their cellular uptake, biodistribution, and clearance behavior. Clearance mechanisms were systematically evaluated both in the blood—*via* immune cells including B cells, T cells, neutrophils, and monocytes—and in the liver, involving hepatocytes, liver sinusoidal endothelial cells (LSECs), and Kupffer cells.

In this study, we aimed to elucidate how macrophage phenotype modulates protein corona formation and reprograms the biological fate of biomimetic nanocarriers (Scheme 1). To this end, we fabricated magnetic silica nanoparticles (SMNs) coated with membranes from M0, M1, or M2 macrophages (designated M0@SMNs, M1@SMNs, and M2@SMNs). We first evaluated their clearance behavior in the bloodstream and liver through in vitro and in vivo models. We then employed nano‐flow cytometry (NanoFCM) to quantify PC‐induced masking of functional membrane ligands (e.g., CD86, CD206) at the single‐particle level. Finally, proteomic profiling, western blotting, and ELISA were used to analyze PC composition, assess complement activation, and evaluate macrophage‐mediated uptake. These findings offer new insights into how macrophage phenotype governs PC formation and immune recognition, providing a rational basis for selecting membrane sources in the development of immune‐evasive nanocarriers.

## 2 Results and Discussion

### 2.1 Preparation and Characterization of M@SMNs with Different Membrane Phenotypes

**FIGURE 1:**
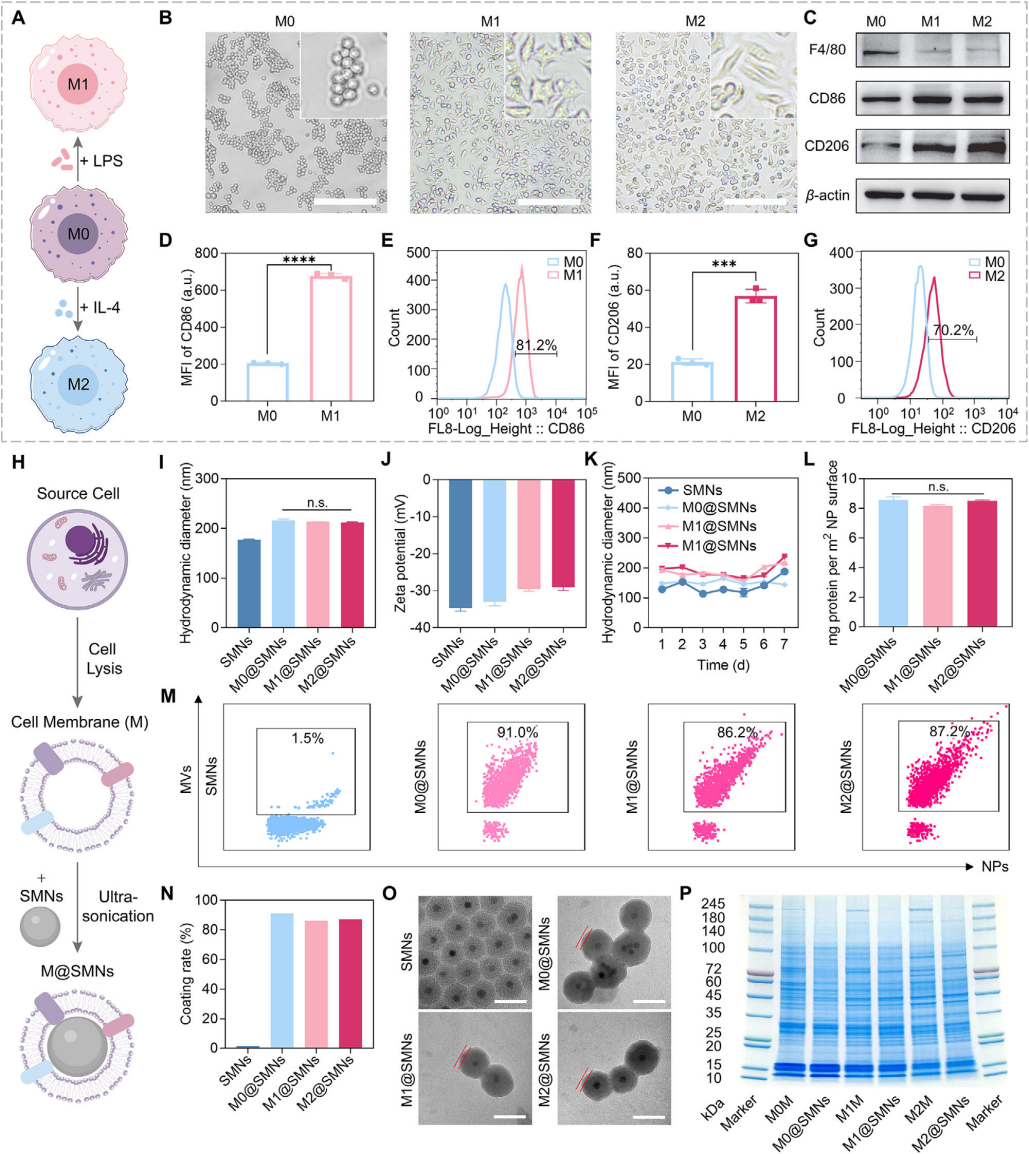
Preparation and characterization of M@SMNs with different membrane phenotypes. (A) Schematic of macrophage polarization. (B) Morphology of macrophages with distinct phenotypes. Scale bar: 50 µm. (C) Western blotting (WB) analysis showing signature protein bands of macrophages with different phenotypes. (D–G) Macrophage polarization efficiency assessed using anti‐CD86 and anti‐CD206 antibodies *via* flow cytometry (FCM). (D, F) Data are presented as mean ± SD (*n* = 3) and statistically analyzed using one‐way ANOVA. ****p < 0.0001. (H) Schematic illustration of the preparation workflow of M@SMNs. (I, J) Hydrodynamic diameter (*D*_h_) and zeta potential of SMNs and M@SMNs. Data are presented as mean ± SD (*n* = 3) and statistically analyzed using one‐way ANOVA. n.s., not significant. (K) Particle size in PBS monitored using DLS over 7 consecutive days. Data are presented as mean ± SD (*n* = 3). (L) Quantification of membrane proteins on M@SMNs using bicinchoninic acid (BCA) assay. Data are presented as mean ± SD (*n* = 3) and statistically analyzed using one‐way ANOVA. n.s., not significant. (M, N) Cell membrane coating rate, defined as the proportion of nanoparticles coated with macrophage membranes relative to the total nanoparticle population, analyzed *via* NanoFCM. (O) Representative transmission electron microscopy (TEM) images of SMNs and M@SMNs. Scale bar: 50 nm. (P) Sodium dodecyl sulfate‐polyacrylamide gel electrophoresis (SDS‐PAGE) analysis of macrophage membranes (M0M/M1M/M2M) and M@SMNs with different phenotypes.

Macrophages exhibit functional heterogeneity and can be categorized into M0 (naïve), M1 (pro‐inflammatory), and M2 (anti‐inflammatory) phenotypes, each defined by characteristic membrane protein signatures. Here, macrophages were polarized toward M1 or M2 states using well‐established induction protocols (Figure 1A) [29, 41]. Morphological alterations following stimulation were consistent with phenotypic differentiation: M1 macrophages exhibited a flattened, “fried egg”‐like morphology while M2 macrophages displayed an elongated, spindle‐shaped appearance (Figure 1B), in agreement with previous reports [42]. Western blot (WB) and flow cytometry (FCM) were employed to evaluate polarization efficiency. As shown in Figure 1C, all subtypes expressed the pan‐macrophage marker F4/80, while CD86 (M1 marker) and CD206 (M2 marker) were selectively upregulated in M1 and M2 cells, respectively [43, 44]. FCM analysis further validated the polarization outcomes: LPS stimulation elevated CD86 expression, yielding an M1 polarization efficiency of 81% (Figure 1D,E), whereas IL‐4 treatment increased CD206 expression, resulting in a 70% M2 polarization rate (Figure 1F,G). These data confirmed the successful production of macrophages with distinct phenotypes.

Silica magnetic nanoparticles (SMNs) were synthesized *via* an inverse microemulsion method [45], yielding particles with a hydrodynamic diameter (*D*_h_) of 177 ± 1 nm and a narrow size distribution as determined by dynamic light scattering (DLS) (Figure S1). Macrophage membranes were isolated through hypotonic lysis and differential centrifugation, followed by ultrasonication to generate membrane vesicles. These vesicles were then fused with SMNs *via* probe ultrasonication to produce macrophage membrane‐coated SMNs (M@SMNs) (Figure 1H). Optimization of the coating process, using M0‐derived membranes as a model, revealed that 9 min of ultrasonication at 100 W led to a smaller particle size without significantly altering the zeta potential (Figure S2). These parameters were subsequently applied for the preparation of M1@SMNs, M0@SMNs, and M2@SMNs.

Macrophage membrane coating increased the hydrodynamic diameter (*D*_h_) of SMNs to 216 ± 3 nm for M0@SMNs, 214 ± 1 nm for M1@SMNs, and 212 ± 2 nm for M2@SMNs (Figure 1I), while maintaining low polydispersity indices (PDIs) (Figure S3). Zeta potentials of the nanoparticles were slightly shifted upon membrane coating, resembling that of the corresponding macrophage‐derived vesicles, which confirmed successful membrane functionalization (Figure 1J; Figure S4) [46, 47]. No significant differences in *D*_h_ were observed among the 3 macrophage phenotypes. Both SMNs and M@SMNs exhibited an excellent colloidal stability over 7 days, with minimal changes in *D*_h_ and zeta potential, and no visible precipitation during storage (Figure 1K; Figure S5). Bicinchoninic acid (BCA) assay revealed comparable membrane protein content across all M@SMNs phenotypes, suggesting a consistent membrane coverage on the nanoparticle surface (Figure 1L).

To further evaluate the coating efficiency, NanoFCM was employed to quantify the proportion of membrane‐coated nanoparticles. Unlike conventional flow cytometry, which is limited to the detection of large particles (>500 nm) with strong fluorescence (typically > 200 fluorescent molecules) [48], NanoFCM allows for multi‐parameter analysis of nanoparticles smaller than 100 nm, commonly applied in extracellular vesicle research [49, 50, 51, 52], and in situ investigation of protein corona formation [53, 54]. This technique provides precise size and fluorescence information at the single‐nanoparticle level through side scattering (SS) and fluorescence intensity (FI) analysis. Hence, it enables a comprehensive evaluation of membrane coating integrity, including both the overall coating rates of nanoparticles and the coverage degree of individual nanoparticles. The average particle size of SMNs measured by NanoFCM was 61 ± 19 nm, while M@SMNs exhibited slightly larger sizes (76 ± 24 nm, 79 ± 28 nm, and 78 ± 26 nm for M0‐, M1‐, and M2‐derived M@SMNs, respectively), consistent with TEM measurements (Figure S6). For detecting membrane coating on SMNs, the SMNs and membrane vesicles were labelled with fluorescein isothiocyanate (FITC) and 1,1'‐dioctadecyl‐3,3,3',3'‐tetramethylindodicarbocyanine perchlorate (DiD), respectively. Fluorescence labelling efficiencies were assessed at the single‐particle level (Figure S7), showing a 92% labelling rate for FITC‐labelled SMNs and 69%, 52%, and 66% for DiD‐labelled membrane vesicles across the 3 phenotypes. M@SMNs were then prepared using FITC‐labelled SMNs and DiD‐labelled vesicles. Co‐localization of FITC and DiD fluorescence on individual particles confirmed successful membrane coating, with more than 85% of total nanoparticles being effectively coated for all phenotypes (Figure 1M,N). The uniformity in membrane coating also led to comparable surface hydrophilicity across M@SMNs, as determined by fluorescence spectroscopy (Figure S8). TEM imaging revealed a typical spherical core–shell structure for M@SMNs, with a membrane coating thickness of approximately 9 nm, consistent with the thickness of a natural cell membrane (Figure 1O) [46]. Further confirmation was obtained using confocal laser scanning microscopy (CLSM). Indeed, SMNs and membranes labelled with FITC (green) and DiD (red), respectively, showed a strong fluorescence overlap, yielding yellow merged signals with Pearson's correlation coefficients of ≈0.9, validating efficient membrane coating across all macrophage phenotypes (Figure S9) [55, 56].

Sodium dodecyl sulfate–polyacrylamide gel electrophoresis (SDS‐PAGE) demonstrated that the protein profiles of M@SMNs closely resembled those of their corresponding source membranes, indicating efficient preservation of membrane proteins during the coating process (Figure 1P). Distinct differences in protein composition were observed among M0‐, M1‐, and M2‐derived membranes. Western blot (WB) analysis further confirmed the successful retention of phenotype‐specific marker proteins, including CD86 on M1@SMNs and CD206 on M2@SMNs (Figure S10). Collectively, these results confirmed the successful fabrication of M@SMNs with high membrane coating efficiency. Despite phenotypic differences in membrane protein composition, all M@SMNs exhibited comparable physicochemical properties, including hydrodynamic diameter and surface hydrophilicity.

### 2.2 In Vitro Macrophage Uptake of M@SMNs with Different Membrane Phenotypes

**FIGURE 2:**
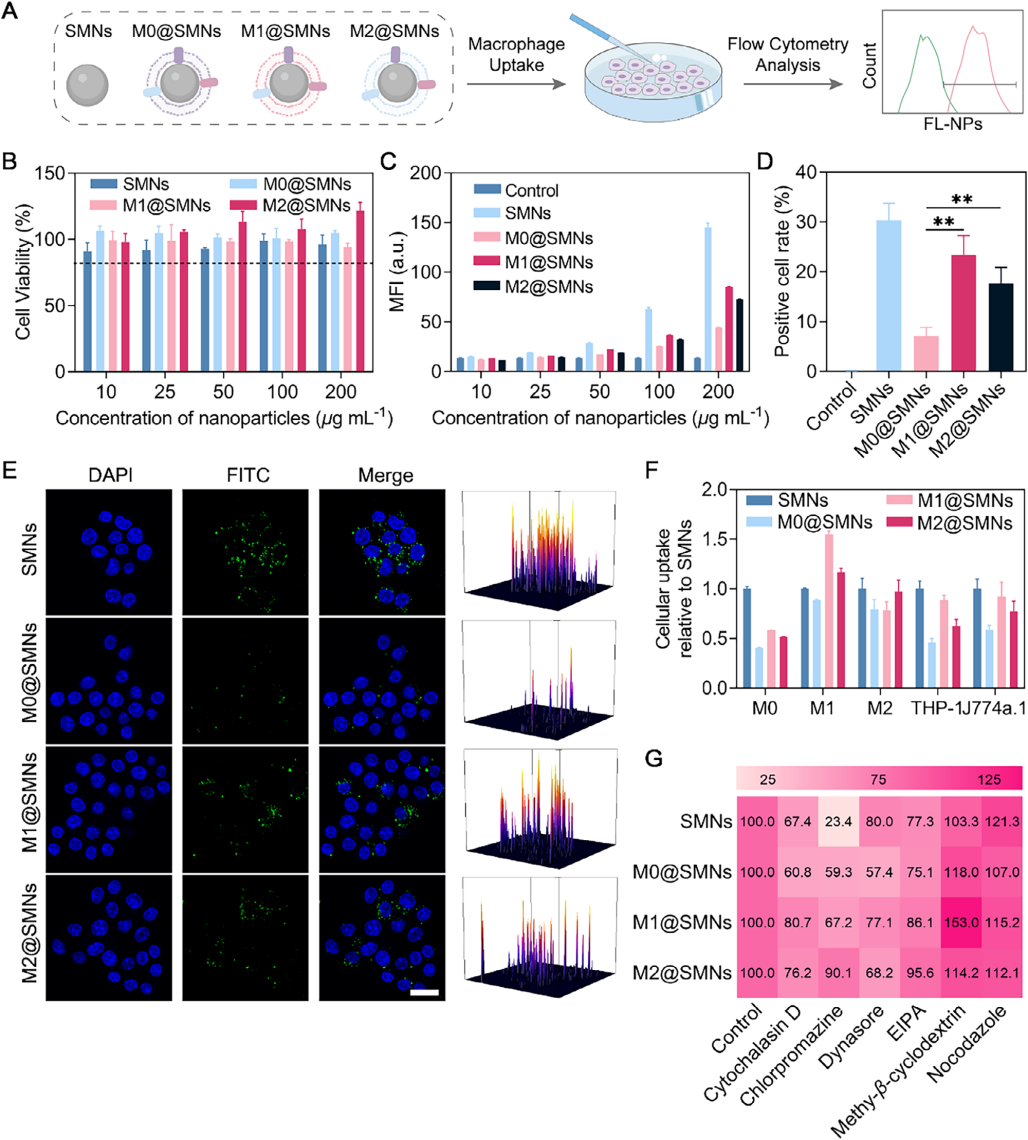
In Vitro Macrophage Uptake of M@SMNs with Different Membrane Phenotypes. (A) Schematic illustration of the macrophage uptake assay. (B) Viability of RAW264.7 cells after treatment with M@SMNs for 24 h. Data are presented as mean ± SD (*n*  =  3). (C) Quantitative analysis of M@SMNs uptake by RAW264.7 cells using FCM. Data are presented as mean ± SD (*n*  =  3). (D) Positive cell rate of M@SMNs uptake by RAW264.7 at 100 µg mL^−1^. Data are presented as mean ± SD and statistically analyzed using Student's *t* test. **p < 0.01. (E) Confocal laser scanning microscopy (CLSM) images showing M@SMNs uptake in RAW264.7 cells and corresponding ImageJ analysis. Nuclei were stained with DAPI, and M@SMNs were labelled with FITC. Scale bar: 20 µm. (F) FCM analysis of M@SMNs uptake by M0‐type RAW264.7, M1‐type RAW264.7, M2‐type RAW264.7, J774a.1, and THP‐1. Data are presented as mean ± SD (*n*  =  3). (G) FCM analysis of endocytic pathways of M@SMNs uptake in RAW264.7 cells. Cells were pretreated with inhibitors (cytochalasin D, chlorpromazine, EIPA, dynasore, methyl‐*β*‐cyclodextrin, or nocodazole) at 37 °C for 1 h.

The cytotoxicity of M@SMNs toward RAW264.7 cells was assessed using the CCK‐8 assay. Across a concentration range of 10–200 µg mL^−1^, cell viability remained above 80%, confirming good biocompatibility of these nanoparticles (Figure 2B) [57]. Cellular uptake of M@SMNs by RAW264.7 macrophages was analyzed using FCM and CLSM (Figure 2A). FCM analysis showed that M@SMNs exhibited consistently lower uptake than uncoated SMNs across all tested concentrations (Figure 2C), indicating that macrophage membrane coating suppresses phagocytosis, likely *via* surface “don't eat me” signals such as CD47 [58, 59]. Among different phenotypes, M0@SMNs exhibited significantly lower uptake than M1@SMNs and M2@SMNs (Figure 2D), a trend further confirmed by CLSM analysis (Figure 2E), suggesting that membrane phenotype may critically influence the in vivo biological fate of M@SMNs.

Under disease conditions, M1 and M2 macrophage populations vary significantly, with M1 macrophages predominantly accumulating in inflammatory tissues and M2 macrophages in tumor microenvironments [60, 61]. Given these distinct tissue distributions, the impact of macrophage phenotypes on nanoparticle uptake was investigated. In M1 macrophages, M1@SMNs exhibited the highest uptake, while M0@SMNs showed the lowest (Figure 2F). Similarly, in M2 macrophages, M2@SMNs were taken up more efficiently, whereas M0@SMNs and M1@SMNs showed lower uptake. These results further underscore the phenotype‐dependency of macrophage uptake of M@SMNs. To investigate the generalizability of these findings across different species and genders, uptake experiments were conducted using THP‐1 macrophages (human‐derived) and J774a.1 macrophage (derived from female mice). Consistent uptake trends were observed across all macrophage types: M1@SMNs showed the highest uptake, followed by M2@SMNs, while M0@SMNs exhibited the lowest uptake. Overall, these findings confirm that M0@SMNs have the lowest uptake, whereas M1@SMNs exhibit the highest uptake (except in M2 macrophages). This is likely due to M0 macrophages representing the resting state found under normal physiological conditions, exhibiting lower immunogenicity, whereas M1 and M2 macrophages, associated with disease states, display enhanced immune activation capabilities [62, 63].

To investigate the influence of phenotypes on the endocytosis pathways in M@SMNs uptake, RAW264.7 cells were pretreated with specific inhibitors, including cytochalasin D, chlorpromazine, EIPA, dynasore, methyl‐*β*‐cyclodextrin, or nocodazole, prior to exposure to M@SMNs. FCM analysis revealed that chlorpromazine significantly reduced SMN uptake (Figure 2G), indicating that clathrin‐mediated endocytosis is the primary pathway [64]. In comparison, M@SMNs are internalized *via* multiple pathways, including clathrin‐mediated endocytosis, caveolin‐mediated endocytosis, and other endocytic mechanisms. Caveolin‐mediated endocytosis was found to predominantly mediate the uptake of M0@SMNs and M2@SMNs, while clathrin‐mediated endocytosis played a more significant role in the uptake of M1@SMNs. These findings suggest that the phenotype of cell membranes influences the endocytic pathways of coated nanoparticles. These distinct endocytic preferences may be attributed to differences in surface markers and membrane properties among M0, M1, and M2 macrophages [23, 65]. M1 macrophages, which exhibit a pro‐inflammatory phenotype with heightened immune activity, express surface markers that favor clathrin‐mediated endocytosis, typically linked to efficient cargo internalization during immune responses [23, 66].

### 2.3 In Vivo Clearance Mechanisms of M@SMNs

**FIGURE 3:**
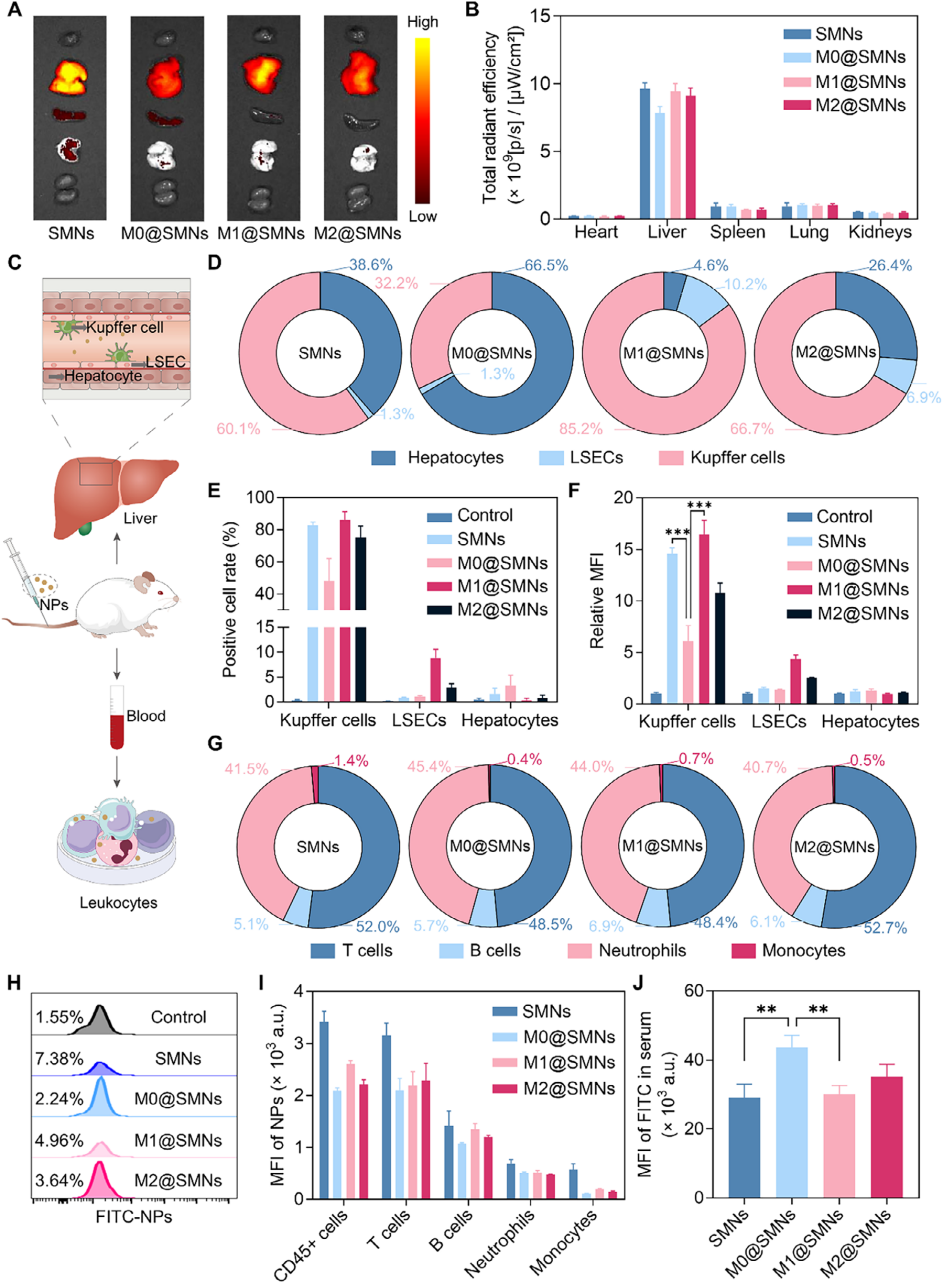
In Vivo Clearance Mechanisms of M@SMNs. (A) Ex vivo fluorescence imaging of major organs collected at 1 h post‐injection. (B) Quantitative fluorescence analysis of major organs. Data are presented as mean ± SD (*n*  =  3). (C) Schematic diagram of liver and blood immune cell uptake experiments. (D) Uptake contribution of each liver cell type to M@SMNs clearance. (E) Positive rate of liver cells involved in M@SMNs uptake. Data are presented as mean ± SD (*n*  =  3). (F) Mean fluorescence intensity (MFI) of M@SMNs uptake in liver cells. Data are presented as mean ± SD (*n*  =  3). (G) Uptake contribution of each blood immune cell type to M@SMNs clearance. (H) Positive rate of blood immune cells involved in M@SMNs uptake. (I) MFI of M@SMNs uptake in blood immune cells. Data are presented as mean ± SD (*n*  =  3). (J) MFI of M@SMNs in mouse serum at 1 h post‐injection. Data are presented as mean ± SD (*n*  =  3) and statistically analyzed using one‐way ANOVA. **p < 0.01.

Based on the observed differences in macrophage uptake among M@SMNs with different phenotypes, we further investigated their in vivo clearance pathways. Prior to animal studies, blood compatibility of M@SMNs was evaluated *via* hemolysis assays. All M@SMNs exhibited hemolysis rates below 5% (Figure S11), confirming their excellent blood compatibility and ensuring the safety of subsequent in vivo experiments [67]. Cy5‐labelled SMNs were administered *via* tail vein injection into BALB/c mice, and biodistribution was monitored using a live imaging system. At 1 h post‐injection, strong fluorescence signals were detected in the liver across all nanoparticle types (Figure 3A,B), indicating that liver was the primary clearance organ for nanoparticles [68, 69]. Notably, M@SMNs showed reduced liver accumulation compared to uncoated SMNs, with M0@SMNs showing the most pronounced reduction (≈29%), followed by M1@SMNs and M2@SMNs (≈9% reduction each), indicative of enhanced immune evasion [70]. Among the phenotypes, M0@SMNs exhibited the lowest liver accumulation, whereas M1@SMNs displayed the highest, reaching ≈1.3‐fold that of M0@SMNs (Figure S12).

To elucidate the hepatic clearance mechanism of M@SMNs following intravenous administration, we analyzed their distribution across major liver cell populations. Liver cells were categorized into parenchymal hepatocytes and non‐parenchymal cells, including Kupffer cells and liver sinusoidal endothelial cells (LSECs). At 1 h post‐injection, liver tissues were dissociated, and nanoparticle uptake was quantified *via* FCM (Figure 3C; Figure S13). To determine each cell population's contribution to total hepatic uptake, we calculated the proportion of total liver‐associated fluorescence attributable to each cell type. Kupffer cells were the dominant contributors, accounting for 85% of total hepatic uptake in the M1@SMNs group. In contrast, their contribution was significantly reduced in the M0@SMNs group (32%), with M2@SMNs showing an intermediate level (67%) (Figure 3D). Flow cytometry further confirmed these findings: M0@SMNs showed the lowest FITC^+^ Kupffer cell percentage (33%), while M1@SMNs and M2@SMNs exhibited 2.4‐fold and 2‐fold higher uptake, respectively (Figure 3F). Notably, M1@SMNs were internalized even more efficiently than uncoated SMNs. LSECs showed a modest but phenotype‐dependent uptake, whereas hepatocytes exhibited minimal nanoparticle internalization across all groups. Interestingly, M0@SMNs displayed slightly elevated hepatocyte association (Figure 3E–F; Figure S14), possibly due to reduced capture by Kupffer cells and deeper penetration into the space of Disse (a liver‐specific interstitial space between hepatocytes and sinusoidal endothelial cells) [71].

In parallel, we evaluated the interaction of M@SMNs with blood immune cells. Flow cytometry analysis revealed low overall nanoparticle uptake (<10% FITC^+^ CD45^+^ cells, Figure 3H; Figure S16). Notably, neutrophils and T cells were the predominant contributors to clearance, together comprising over 90% of FITC^+^ immune cells (Figure 3G). Among all formulations, M0@SMNs exhibited the lowest immune cell uptake, as evidenced by significantly reduced MFI across all cell subsets (Figure 3I), indicating a superior immune evasion. Consistently, M0@SMNs exhibited the highest blood retention at 1 h post‐injection (Figure 3J), highlighting their enhanced stability and prolonged circulation in vivo.

### 2.4 Analysis of PC Formation and Its Impact on Cellular Uptake

The in vivo studies have shown that hepatic and blood clearance of M@SMNs are highly dependent on the macrophage phenotype used for membrane coating. However, given the complexity of the in vivo environment—particularly the high concentration of serum proteins—nanoparticles inevitably acquire a PC upon systemic exposure, which can markedly influence their biological fate [72, 73, 74]. Therefore, beyond the intrinsic properties of the cell membrane, the effect of PC formation should also be considered. It is important to acknowledge that serum and plasma can produce partially distinct PC profiles, primarily due to differences in coagulation‐related proteins that are depleted during serum preparation [75]. Nevertheless, serum remains extensively used in PC research and provides a reliable framework for comparative analyses when experimental conditions are maintained consistently across groups [36, 76, 77, 78, 79, 80].

**FIGURE 4:**
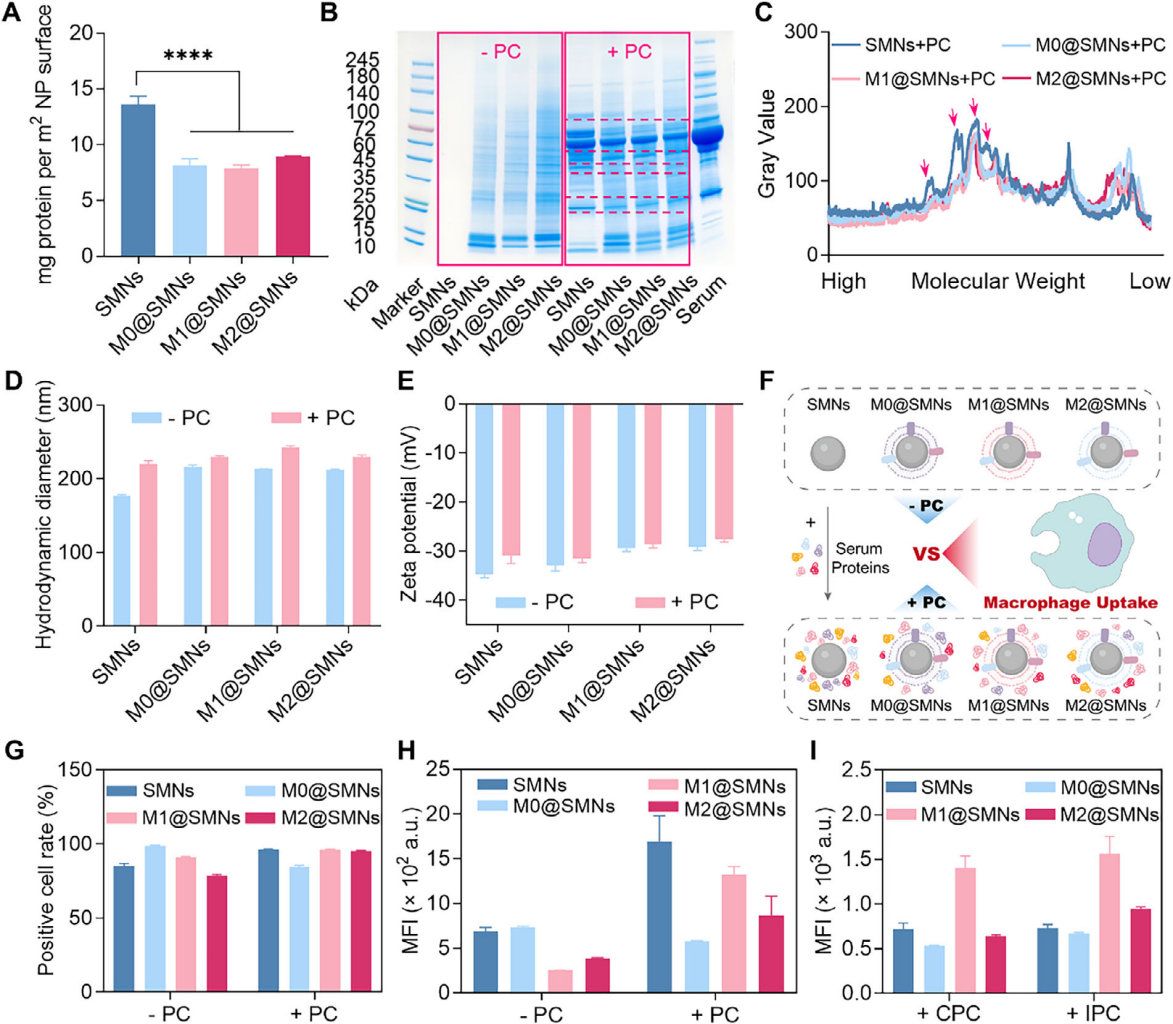
Analysis of PC Formation and Its Impact on Macrophage Uptake. (A) Quantification of serum proteins adsorbed onto the nanoparticle surface using the BCA assay. Data are presented as mean ± SD (*n* = 3) and analyzed by one‐way ANOVA. ****p < 0.0001. (B) SDS‐PAGE analysis showing the protein profiles adsorbed on nanoparticles before and after serum incubation. (C) Densitometric analysis of protein bands in panel (B) using ImageJ, with significant changes indicated by red arrows. (D, E) Hydrodynamic diameter (*D*_h_) and zeta potential of nanoparticles before and after serum incubation. Data are presented as mean ± SD (*n* = 3). (F) Schematic illustration of macrophage uptake of M@SMNs with or without a PC. (G) Percentage of RAW264.7 cells positive for nanoparticle uptake. Data are presented as mean ± SD (*n* = 3). (H) FCM analysis of RAW264.7 uptake of M@SMNs before and after serum incubation. Data are presented as mean ± SD (*n* = 3). (I) FCM analysis of RAW264.7 uptake of M@SMNs incubated with different types of serum. CPC: cancer‐associated PC (breast cancer serum); HLC: hyperlipidemic‐associated PC (hyperlipidemic serum). Data are presented as mean ± SD (*n* = 3).

To quantify the serum protein adsorption on M@SMNs, we performed BCA assays following incubation of nanoparticles with mouse serum (Figure 4A). All M@SMNs formulations exhibited detectable protein adsorption, although to a significantly lesser extent than uncoated SMNs. Specifically, the amounts of adsorbed proteins on M0@SMNs, M1@SMNs, and M2@SMNs were 60%, 59%, and 66% relative to pristine SMNs, respectively. No significant differences were observed among the 3 phenotypes, indicating that while membrane coating reduces protein adsorption, it does not completely eliminate PC formation. SDS‐PAGE analysis further confirmed the presence of PC on M@SMNs, as evidenced by enhanced intensity of protein bands following serum exposure (Figure 4B). The composition of adsorbed proteins on M@SMNs differed from that on SMNs, with M@SMNs exhibiting a marked reduction in mid‐molecular‐weight protein bands (Figure 4C). In addition, protein adsorption led to slight increase in the hydrodynamic diameter, with minimal influence of zeta potential (Figure 4D–E).

To determine whether PC formation alters macrophage recognition, we assessed the uptake of M@SMNs by RAW264.7 cells before and after serum incubation, using FCM (Figure 4F). In the absence of a PC, M0@SMNs exhibited the highest cellular uptake, whereas M1@SMNs and M2@SMNs showed lower internalization levels (Figure 4G,H). Upon PC formation, uptake by macrophages increased significantly across all M@SMNs formulations. Notably, the uptake of M1@SMNs and M2@SMNs increased by 2.3‐fold and 1.5‐fold, respectively, relative to M0@SMNs. Furthermore, M@SMNs were incubated with breast cancer serum or hyperlipidemic serum to form pathology‐associated protein coronas before being exposed to RAW264.7 macrophages. Remarkably, M1@SMNs with preformed coronas exhibited even higher uptake than uncoated SMNs (Figure 4I), indicating that disease‐derived coronas can enhance macrophage recognition and internalization of inflammatory phenotype‐coated particles. These findings highlight the critical role of protein corona in reshaping the phenotype‐dependent recognition of M@SMNs by macrophages, and suggest that in certain phenotypes, PC formation may override the immune‐evasive effects conferred by membrane coating.

### 2.5 Impact of PC Formation on Membrane Protein Accessibility of M@SMNs

To evaluate how PC formation affects the accessibility of membrane proteins on M@SMNs, we first employed NanoFCM to quantitatively assess the amount of serum protein adsorption. FITC‐labelled M@SMNs were incubated with Cy5‐labelled human serum proteins and analyzed by NanoFCM (Figure S17). Human serum was selected in this experiment to enhance clinical relevance. Following serum incubation, a notable rightward shift in particle size distribution was observed (Figure S18), indicating increased particle size resulting from PC formation. At the overall level, quantitative analysis of protein coverage rates revealed that 52% of SMNs adsorbed a detectable amount of serum proteins, compared to a coverage rate of 42% for M0@SMNs, 44% for M1@SMNs, and 42% for M2@SMNs, suggesting that membrane coating was reduced but did not fully prevent PC formation. At the single‐particle level, SMNs exhibited the highest proportion of particles with high protein coverage degrees (50%–100%) at 34%, followed by M1@SMNs (37%), M2@SMNs (32%), and M0@SMNs (21%) (Figure S19a). In contrast, SMNs showed the lowest fraction of particles with low coverage degrees (0%–25%), accounting for only 25%, compared to 28% for M1@SMNs, 34% for M2@SMNs, and 37% for M0@SMNs (Figure S19b). Collectively, these findings confirm that serum incubation leads to PC formation on all particle types and that particle surface coverage is influenced by membrane phenotype. Notably, M0@SMNs and M2@SMNs showed overall lower surface protein coverage than SMNs and M1@SMNs, highlighting phenotype‐dependent differences in corona formation. These results suggest that M0 and M2 membrane coatings are more effective in minimizing serum protein deposition.

**FIGURE 5:**
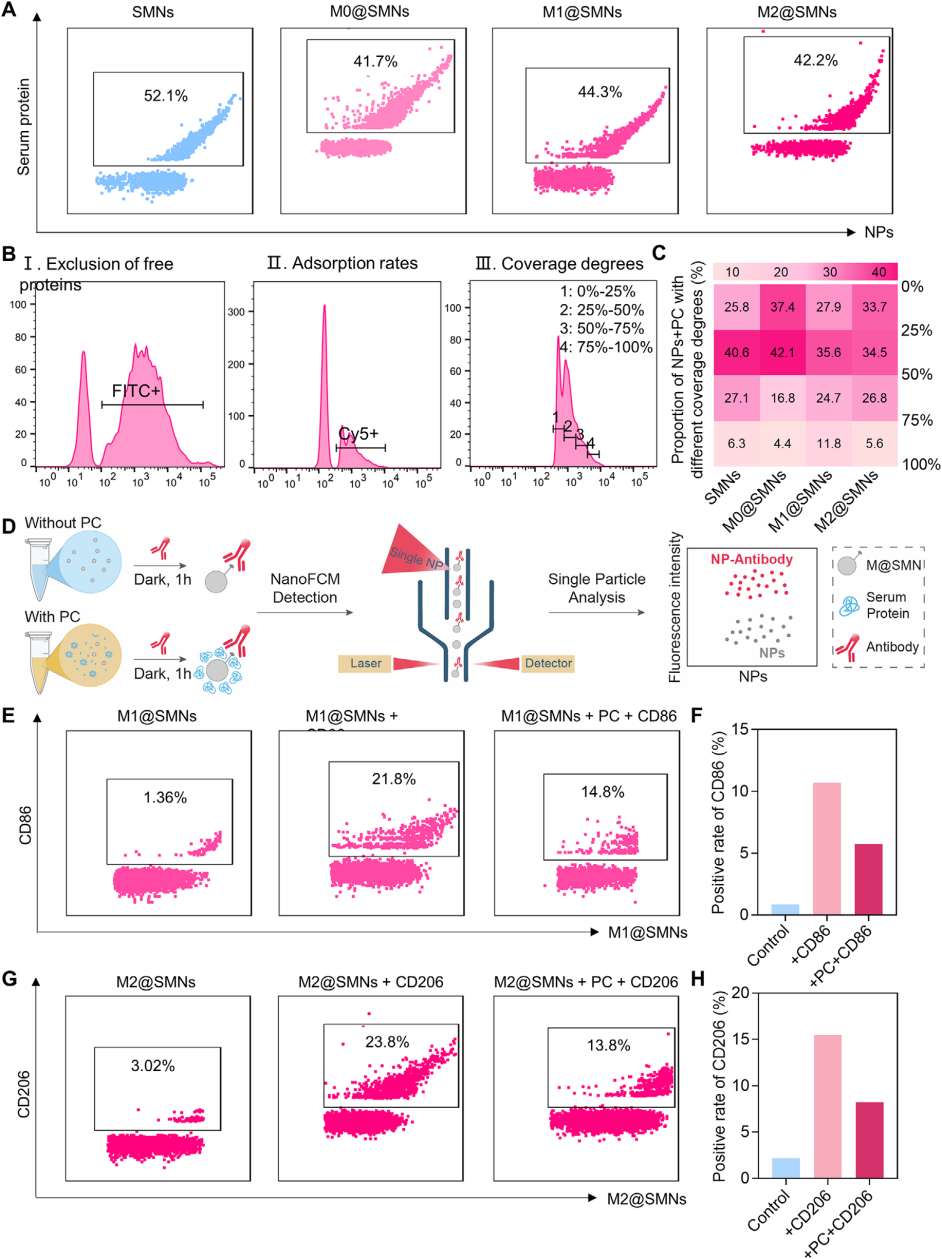
Impact of PC Formation on Membrane Protein Accessibility of M@SMNs. (A) Percentage of nanoparticles with detectable human serum protein adsorption, defined as the protein coverage rate (% of total particles). (B) NanoFCM‐based gating strategy for evaluating the surface protein coverage degree (% of particle surface area) at the single‐particle level. Fluorescence histograms were divided into four gated regions corresponding to coverage degrees of 0–25%, 25–50%, 50–75%, and 75–100%. (C) Distribution of M@SMNs with different surface protein coverage degrees (0%–100%). (D) Schematic representation of NanoFCM‐based single‐particle analysis to assess the masking of membrane proteins by PC formation on M@SMNs. (E and G) Representative scatter plots showing antibody binding to phenotype‐specific markers—CD86 on M1@SMNs (E) and CD206 on M2@SMNs (G)—before and after human serum incubation. (F and H) Quantitative comparison of antibody‐binding rates for M1@SMNs and M2@SMNs before and after incubation with human serum.

To further assess the extent to which PC formation masks functional membrane proteins on M@SMNs, NanoFCM was employed to quantify the accessibility of phenotype‐specific surface markers—CD86 for M1@SMNs and CD206 for M2@SMNs—using fluorescently labelled antibodies (Figure 5D). Prior to serum incubation, antibody binding rates were 22% for CD86 on M1@SMNs and 24% for CD206 on M2@SMNs, confirming the successful retention of phenotype‐specific membrane proteins. However, after incubation with human serum, the corresponding binding rates decreased to 15% and 14%, respectively, indicating that a substantial portion of these ligands became inaccessible due to PC coverage (Figure 5E,G). The relative masking efficiencies were calculated to be 32% for M1@SMNs and 42% for M2@SMNs, reflecting a phenotype‐dependent shielding effect (Figure 5F,H). Collectively, these results demonstrate that PC formation compromises the accessibility of key membrane proteins, which may undermine the intended biological functions of M@SMNs.

### 2.6 Analysis of PC Composition and Complement Activation

**FIGURE 6:**
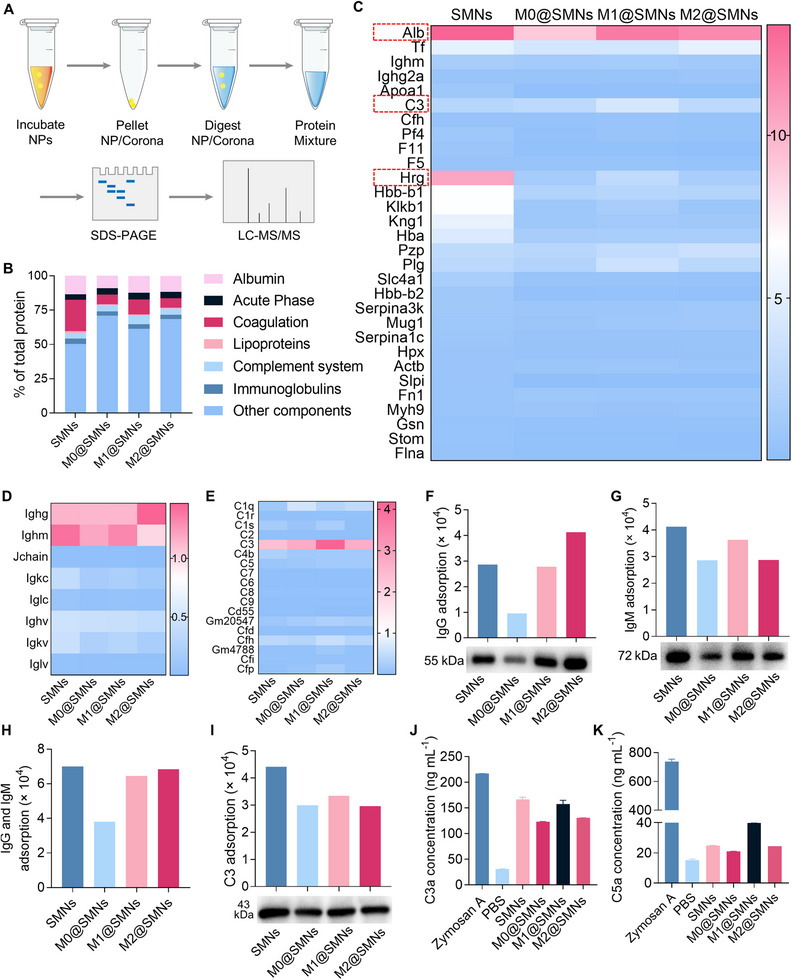
Analysis of PC Composition and Complement Activation. (A) Schematic diagram of the LC‐MS/MS workflow for PC profiling. (B) Classification of PC components identified via LC‐MS/MS. (C) Heatmap depicting the top 30 most abundant proteins enriched in the PC. (D) Heatmap showing the distribution of immunoglobulin subtypes in the PC. (E) Heatmap of complement proteins identified in the PC. (F, G) Western blot analysis of IgG and IgM adsorption on SMNs and M@SMNs, with band intensity quantified using ImageJ. (H) Quantification of immunoglobulins (IgG and IgM) adsorbed on nanoparticle surfaces using ImageJ software. (I) Western blot analysis of complement C3 adsorption levels, with densitometry quantification. (J, K) Quantification of complement activation markers (C3a and C5a) by ELISA in human serum following nanoparticle incubation. PBS and zymosan (3 mg mL^−^^1^) were used as negative and positive controls, respectively. Data are presented as mean ± SD (*n* = 3).

To investigate phenotype‐dependent differences in PC composition, adsorbed proteins were eluted from M@SMNs using 2 wt% SDS (Figure 6A). LC‐MS/MS analysis revealed that membrane coating notably reduced the adsorption of medium‐sized proteins (60–80 kDa, Figure S19A) and positively charged proteins (Figure S19B), compared to uncoated SMNs. Although the types of adsorbed proteins were largely conserved across SMNs, M0@SMNs, M1@SMNs, and M2@SMNs, their relative abundances varied markedly (Figure 6B). Notably, M0@SMNs exhibited the lowest levels of coagulation‐related proteins and immunoglobulins, indicating reduced immune recognition potential. Among the top 30 enriched proteins, serum albumin—a dominant component of the PC—was significantly decreased upon membrane coating, with the lowest abundance observed on M0@SMNs (Figure 6C). Interestingly, histidine‐rich glycoprotein (Hrg), a multifunctional opsonin implicated in immune clearance and cell adhesion [81, 82], was substantially enriched on M1@SMNs but nearly absent on M0@SMNs, which may partially explain the increased phagocytic uptake of M1@SMNs by macrophages.

**SCHEME 2:**
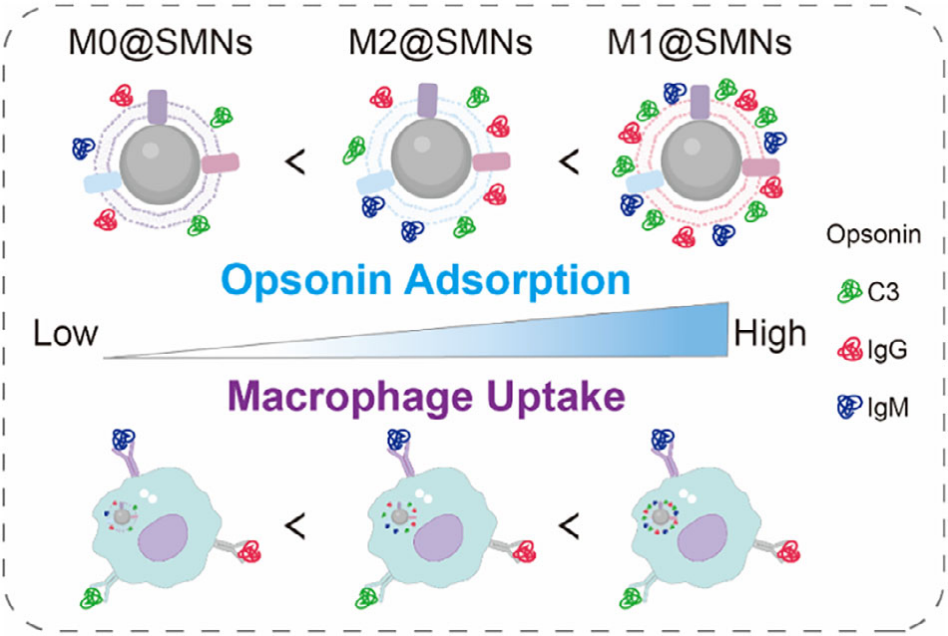
Schematic illustration of phenotype‐dependent opsonin adsorption and macrophage uptake of M@SMNs.

Complement proteins and immunoglobulins, as key immune opsonins, play pivotal roles in nanoparticle recognition and clearance [83, 84, 85]. Proteomic profiling revealed substantial phenotype‐dependent differences in their adsorption across M@SMNs (Figure 6D,E). Western blot analysis further confirmed that M0@SMNs exhibited the lowest levels of adsorbed C3, IgG, and IgM (Figure 6F–I), whereas M1@SMNs displayed the highest adsorption, consistent with their pro‐inflammatory membrane phenotype. Given the critical role of complement activation in opsonization, we quantified the generation of C3a and C5a fragments by ELISA. Among all groups, M0@SMNs triggered the lowest C3a and C5a levels in both human and murine sera (Figure 6J,K; Figure S20), indicating attenuated complement activation. Taken together, the reduced enrichment of immune opsonins and lowered complement cascade activation contribute to the superior immune stealth and lower hepatic clearance of M0@SMNs (Scheme 2). The phenotype‐dependent opsonin adsorption observed in this study likely arises from polarization‐driven remodeling of macrophage membrane architecture. Macrophage activation alters membrane protein composition, glycosylation, and surface charge, thereby reshaping immune recognition at nano–bio interfaces [86, 87]. M1‐derived membranes, enriched in pro‐inflammatory markers, may present stronger immune recognition cues that favor complement deposition and immunoglobulin binding, promoting opsonization and rapid clearance [88]. In contrast, M0 membranes retain a comparatively quiescent surface phenotype with reduced exposure of complement‐triggering motifs, resulting in diminished opsonin recruitment and attenuated hepatic sequestration, consistent with the concept that nanoparticle fate is primarily dictated by the acquired protein corona [89]. M2 membranes exhibited an intermediate profile, potentially reflecting their immunoregulatory characteristics and moderated serum protein interactions [90]. Collectively, these findings support a phenotype–corona–clearance relationship, identifying source cell phenotype as a critical yet underrecognized parameter for engineering biomimetic nanocarriers with programmable immune fates.

These findings highlight the translational potential of phenotype‐specific macrophage membranes. M0 membranes, owing to their reduced opsonin adsorption and attenuated immune activation, are promising for constructing nanocarriers that require prolonged systemic circulation and effective tumor targeting. In contrast, M1 membranes, which exhibit elevated opsonin recruitment and rapid immune clearance, may be better suited for cancer immunotherapy where immunostimulatory effects are advantageous. M2 membranes, defined by their immunoregulatory phenotype, could offer therapeutic value in inflammatory and autoimmune disorders such as rheumatoid arthritis. Nevertheless, further studies are required to evaluate the long‐term stability, immunogenicity, and scalability of these biomimetic systems for clinical translation.

Despite the promising biological performance observed in this study, several challenges remain for the clinical translation of membrane‐coated nanoparticles. The preparation of these biomimetic systems involves multiple steps and biological materials, which may introduce variability and complicate large‐scale manufacturing. In addition, regulatory considerations associated with cell‐derived components warrant careful evaluation. Nevertheless, rapid advances in membrane vesiculation technologies, scalable extrusion strategies, and biomimetic engineering are progressively improving the reproducibility and manufacturability of such platforms. Importantly, the present work is intended primarily as a mechanistic and conceptual advance that elucidates how source cell phenotype governs protein corona evolution and immune fate. These insights provide a rational framework for the future design of biomimetic nanocarriers with improved translational potential.

## 3 Conclusions

This study systematically elucidates the phenotype‐dependent clearance behaviors of macrophage membrane‐coated SMNs and the mechanistic contribution of the protein corona (PC). Among the 3 phenotypes, M0@SMNs exhibited the most pronounced immune evasion, evidenced by markedly reduced macrophage uptake in vitro and prolonged blood retention in vivo compared with M1@SMNs and M2@SMNs. Biodistribution analyses further revealed substantially decreased Kupffer cell sequestration for M0@SMNs, indicating attenuated hepatic clearance. Despite membrane coating, all M@SMNs formed a PC upon serum exposure, which modulated their macrophage recognition. NanoFCM single‐particle analysis revealed heterogeneous PC coverage and ∼40% reduction in detectable membrane ligands (CD86/CD206) due to PC masking. Mechanistic investigations showed that M0@SMNs adsorbed the lowest levels of immune opsonins (C3, IgG, IgM) and elicited minimal complement activation, collectively contributing to their superior stealth properties. Together, these findings underscore that the source cell phenotype dictates PC composition and clearance behavior, and establish M0 macrophages as the preferred source for extending systemic circulation relative to M1 and M2 subtypes.

## 4 Experimental Section

### 4.1 Materials

Cyclohexane (≥99%), Igepal CO‐520 (≥99%), aqueous ammonia solution (NH_3_·H_2_O, 25%–28%), fluorescein isothiocyanate (FITC), tetraethyl orthosilicate (TEOS, ≥99%), and 3‐aminopropyltriethoxysilane (APTES, ≥99%) were purchased from Aladdin Biochemical Technology Co., Ltd. (Shanghai, China). Recombinant murine interleukin‐4 (IL‐4) was obtained from Peprotech (New Jersey, USA), and lipopolysaccharide (LPS) from Sigma‐Aldrich (Missouri, USA). Penicillin–streptomycin solution (100×) was purchased from Cytiva (Washington, USA). Dulbecco's Modified Eagle Medium (DMEM), fetal bovine serum (FBS), and trypsin–EDTA (0.25%) were obtained from Gibco (California, USA). APC‐labelled anti‐mouse CD86 and CD206 antibodies, TruStain FcX PLUS (anti‐mouse CD16/32), anti‐C3, anti‐IgG antibodies, and staining buffer were purchased from BioLegend Inc. (California, USA). Fixation/Permeabilization Kit was from BD Pharmingen (New Jersey, USA). Phosphate‐buffered saline (PBS), 4',6‐diamidino‐2‐phenylindole (DAPI), and 4% paraformaldehyde were purchased from Solarbio Science & Technology Co., Ltd. (Beijing, China). Coomassie brilliant blue staining solution, DiD, Cell Counting Kit‐8 (CCK‐8), serum‐free cryopreservation medium, antibody dilution buffers, western transfer buffer, and QuickBlock blocking buffer were purchased from Beyotime Biotechnology Co., Ltd. (Shanghai, China). Cyanine5 NHS Ester (Cy5‐SE) was obtained from MedChemExpress (Shanghai, China). BCA Protein Assay Kit was provided by Nanjing Jiancheng Bioengineering Institute (Nanjing, China). Protease inhibitor cocktail was from Selleck (Shanghai, China). 4–20% precast polyacrylamide gels, running buffer, protein markers, and ECL detection kits were supplied by Yeasen Biotechnology Co., Ltd. (Shanghai, China). Goat anti‐rabbit IgG H&L (HRP‐conjugated, #abs20163) was obtained from Absin (Shanghai, China). ELISA kits for human and mouse C3a/C5a, as well as antibodies against CD86, CD206, F4/80, and β‐actin were purchased from Wuhan Fine Biotechnology Co., Ltd. (Wuhan, China). All reagents were used without further purification unless otherwise specified. Milli‐Q water was used throughout the experiments for solution preparation and washing steps.

### 4.2 Methods

#### 4.2.1 Animals and Ethics Statement

Female BALB/c mice (20‐25 g) were purchased from Pengyue Laboratory Animal Breeding Co., Ltd. (Jinan, China). Mice were housed in constant environmental conditions (room temperature, 25 ± 1°C, relative humidity, 40%–70%, and a 12 h light–dark cycle). Mice were access to food and water free. All animal experiments were approved by the Institutional Animal Care and Ethics Committee of Ocean University of China (ethical approval number: OUC‐SMP‐2024‐02‐01).

#### 4.2.2 Cell Culture

Cell lines (RAW264.7, THP‐1, and J774a.1) were obtained from Cell Bank of the Chinese Academy of Sciences (Shanghai, China). All cells were cultured at 37°C in a humidified atmosphere with 5% CO_2_ and maintained in DMEM supplemented with 10% FBS and 1% penicillin–streptomycin solution.

#### 4.2.3 Mouse Serum

Mouse serum was collected via retro‐orbital bleeding. Female BALB/c mice were anesthetized with isoflurane, and 0.5–1.0 mL of whole blood was drawn from the retro‐orbital sinus using sterile, heparin‐free capillary tubes. The blood was transferred into sterile 1.5 mL centrifuge tubes and left to clot at room temperature for 30 min. Samples were then centrifuged at 3000 ×g for 10 min at 4°C. The resulting serum was collected, aliquoted, and stored at –80°C until use.

#### 4.2.4 Human Serum

Whole blood from 10 apparently healthy donors was collected in clot‐activator tubes and allowed to clot at room temperature, following the Declaration of Helsinki, with informed consent and approval from the Ethics Committee of Ocean University of China (No. OUC‐HM‐2022‐01). Samples were centrifuged at 1,700 ×g for 5 min to remove the clot, and the resulting serum was pooled and stored at −80°C.

#### 4.2.5 RAW264.7 Activation and Characterization

##### 4.2.5.1 RAW264.7 Activation

RAW264.7 macrophages were activated according to established protocols [41]. To induce M1 polarization, cells were stimulated with 100 ng/mL lipopolysaccharide (LPS) for 24 h. For M2 polarization, cells were treated with 40 ng/mL interleukin‐4 (IL‐4) for 48 h. Cellular morphology and phenotypic changes were observed using an optical microscope (Nikon Eclipse Ts2, Japan).

##### 4.2.5.2 Flow Cytometry (FCM) Analysis

Activated RAW264.7 macrophages were identified by FCM. Briefly, M1‐ or M2‐polarized cells were harvested and incubated with 0.25 µg of Fc receptor‐blocking reagent (anti‐mouse CD16/32) for 10 min to minimize nonspecific binding. For M1 macrophages, cells were stained with 0.25 µ*g* APC‐conjugated anti‐CD86 antibody for 20 min at room temperature. For M2 macrophages, cells were fixed and permeabilized using 250 µ*L* of fixation/permeabilization reagent, followed by intracellular staining with 0.5 µ*g* APC‐conjugated anti‐CD206 antibody for 20 min. After staining, cells were washed 3 times with staining buffer (350 ×g, 5 min each) to remove unbound antibodies. Samples were analyzed using a MoFlo XDP flow cytometer (Beckman Coulter, USA), and data were processed with FlowJo software (TreeStar, USA).

##### 4.2.5.3 Western Blotting Analysis

WB analysis was performed to evaluate the expression of macrophage membrane proteins. M0, M1, and M2 macrophages were lysed on ice for 20 min using RIPA lysis buffer (containing 1 mM PMSF). The lysed samples were centrifuged at 17,970 ×g and 4°C for 10 min to collect the supernatant for further analysis. Protein concentrations were determined using a BCA assay kit, after which the samples were mixed with loading buffer at a 4:1 ratio and heated at 95°C for 10 min to denature the proteins. Proteins were separated using 8% SDS‐PAGE gels under constant 120 V electrophoresis and subsequently transferred onto nitrocellulose (NC) membranes at 110 V. The membranes were blocked with rapid blocking buffer, followed by incubation with a series of primary antibodies (anti‐*β*‐actin, anti‐CD86, anti‐CD206, or anti‐F4/80) at 4°C overnight. On the following day, membranes were incubated with the corresponding secondary antibodies at room temperature for 1 h. Finally, protein bands were visualized using a gel imaging system (Tanon 4600, Tianneng Life Science Co., Ltd., China).

#### 4.2.6 Preparation of Macrophage Membrane‐Coated Nanoparticles

##### 4.2.6.1 Synthesis of Fe_3_O_4_ Nanoparticles

Fe_3_O_4_ nanoparticles were synthesized following a previously reported method [45]. Briefly, 2 mmol of FeCl_3_·6H_2_O was dissolved in 6 mL of H_2_O, followed by adding 8 mL of ethanol, 14 mL of hexane, and 1.9 mL of oleic acid. The mixture was stirred at room temperature for 30 min, after which 0.24 g of NaOH was added, and the reaction proceeded in a closed vessel at 70°C for 4 h. The resulting solution was separated using a separatory funnel, and the organic layer containing the Fe(oleate)_3_ complex was collected, washed three times with deionized water, and heated at 80°C overnight to remove the hexane. The Fe(oleate)_3_ precursor was dispersed in a mixture of 0.32 mL of oleic acid and 12.5 mL of 1‐octadecene, degassed under N_2_ at room temperature for 30 min, and heated to 320°C for 30 min under N_2_ flow. The resulting Fe_3_O_4_ nanoparticles were precipitated with ethanol, collected by centrifugation, and purified through five precipitation‐redispersion cycles. The precipitate was dispersed in cyclohexane at a concentration of 1 mg mL^−1^ for subsequent use.

##### 4.2.6.2 Preparation of Silica Magnetic Nanoparticles (SMNs)

A reverse microemulsion method was used to prepare SMNs [45]. Briefly, 0.5 g of Igepal CO‐520 and 4 mL of Fe_3_O_4_ solution (1 mg mL^−1^ in cyclohexane) were dispersed in 7 mL of cyclohexane under continuous stirring. Subsequently, 0.1 mL of NH_3_·H_2_O was added, and the solution was stirred for 10 min until it became transparent. TEOS was then introduced using the equivalently fractionated drop method (adding 25 µ*L* of TEOS every 12 h, 3 times). The synthesized SMNs were purified by eluting with ethanol and centrifuging five times at 11,140 ×g to remove residual reagents. Finally, the nanoparticles were redispersed in deionized water using ultrasonication for further use.

FITC‐labelled SMNs were synthesized by incorporating FITC‐APTES into the silica precursors. First, 2 mg of FITC was dissolved in 2 mL of methanol, followed by the addition of 11.37 µL of APTES under continuous stirring at 50 ×g. The mixture was stirred at room temperature for 48 h, yielding FITC‐APTES. Subsequently, 30 µL of the FITC‐APTES solution was combined with TEOS to serve as the silica precursor. The preparation and purification steps for FITC‐labelled SMNs were identical to those for SMNs.

##### 4.2.6.3 Macrophage Membrane Extraction

Macrophages were washed with PBS to remove dead cells and debris, resuspended in PBS, and centrifuged at 50 ×g for 3 min to collect the cell pellet. The pellet was resuspended in 10 mL of hypotonic lysing buffer (20 mM Tris‐HCl, 10 mM KCl, 2 mM MgCl_2_, pH 7.4) supplemented with 1% protease inhibitors and lysed at 4°C for 2 h. The lysate was further disrupted using an ultrasonic cell disruptor (JY92‐IIN, Ningbo Xinzhi Biotechnology Co., Ltd., China) at 100 W for 6 min (2 s on/10 s off).

Cell membranes were isolated through differential centrifugation. First, the lysed cell suspension was centrifuged at 250 ×g for 5 min to remove unbroken cells and large debris, and the resulting supernatant was collected. This supernatant was further centrifuged at 1,442 ×g for 15 min to remove the organelles (as a pellet). The final supernatant was centrifuged at 13,492 ×g for 30 min to pellet the cell membranes, which were washed three times with PBS. The purified membranes were resuspended in PBS, quantified using a BCA assay kit, and stored at ‐80°C for subsequent experiments.

##### 4.2.6.4 Preparation of Macrophage Membrane‐Coated SMNs (M@SMNs)

Cell membranes were ultrasonicated at 100 W for 9 min (2 s on/4 s off) in an ice bath to generate membrane vesicles. These vesicles were mixed with SMNs at a 1:1 mass ratio and further ultrasonicated at 100 W for 9 min (2 s on/4 s off) to produce M@SMNs. To optimize membrane coating efficiency, ultrasonication duration (3 to 21 min) and power (30 to 400 W) were varied.

#### 4.2.7 Characterization of SMNs and M@SMNs

##### 4.2.7.1 Dynamic Light Scattering (DLS) Characterization

The hydrodynamic diameter (*D*_h_) and zeta potential of the nanoparticles were measured using a Zetasizer Nano‐ZS ZEN3600 (Malvern Instruments Co., Ltd., UK). Stability of nanoparticles was evaluated in PBS for one week.

##### 4.2.7.2 Transmission Electron Microscopy (TEM) Characterization

The morphology of nanoparticles was characterized using TEM (JEM‐2100EX, JEOL Ltd., Japan) following negative staining with a 1% uranyl acetate solution. Briefly, a copper grid was immersed in the nanoparticle suspension and allowed to adsorb the sample for 3 min. The grid was then carefully removed, and excess liquid was blotted off with absorbent paper. Subsequently, a drop of 1% uranyl acetate was applied to the copper grid and allowed to stain for 3 min. Afterwards, the excess stain was removed, and the grid was dried in air prior to TEM imaging.

##### 4.2.7.3 Confocal Laser Scanning Microscopy (CLSM) and Nano Flow Cytometry (NanoFCM) Characterization

CLSM was used to analyze the fluorescence colocalization of cell membrane and nanoparticles [55]. Briefly, the cell membranes were labelled with DiD by incubating with 50 µ*L* of 1 mg mL^−1^ DiD for 2 h in 4°C, while SMNs were labelled with FITC as described in section 4.2.6. The dual‐fluorescence‐labelled nanoparticles were visualized using CLSM (Zeiss LSM 800, Leica Microsystems, Germany). The nanoparticles were further analyzed using a Nano Flow Cytometry (NanoFCM) system (Xiamen Fuliu Biotechnology Co., Ltd., China).

##### 4.2.7.4 Sodium Dodecyl Sulfate‐Polyacrylamide Gel Electrophoresis (SDS‐PAGE) Analysis

For SDS‐PAGE analysis, cell membrane proteins were desorbed from M@SMNs using 2 wt% SDS solution. The samples were mixed with loading buffer and boiled for 10 min. Proteins were separated using 4%–20% Precast Protein Plus Gel. The gel was electrophoresed at 150 V for 45 min until the loading buffer band reached the bottom of the gel. The gel was then stained with Coomassie Brilliant Blue at room temperature overnight, washed with distilled water, and photographed.

#### 4.2.8 In Vitro Macrophage Uptake of M@SMNs with Different Membrane Phenotypes

##### 4.2.8.1 Cell Viability Assay

The CCK‐8 assay was carried out to assess the cell viability after treatment with SMNs and M@SMNs. RAW264.7 cells were seeded in 96‐well plates at a density of 1 × 10^4^ cells per well and incubated at 37°C for 24 h. Then, the cells were treated with various concentrations (0‐200 µg mL^−1^) of SMNs or M@SMNs for 24 h. After treatment, 90 µL of fresh medium and 10 µL of CCK‐8 reagent were added to each well, and the cells were incubated for an additional 4 h. Finally, absorbance at 450 nm was measured using a multimode microplate reader. Cell viability was calculated using the formula:

Cellviability(%)=As/Ac×100%

where A_s_ was the absorbance of the experimental well and A_c_ was the absorbance of the control well.

##### 4.2.8.2 CLSM and FCM

RAW264.7 (M0), LPS‐polarized RAW264.7 (M1), IL‐4‐polarized RAW264.7 (M2), THP‐1, and J774a.1 cells were seeded in 6‐well plates at a density of 5 × 10^5^ cells/well and incubated for 24 h. Subsequently, cells were treated with FITC‐labelled SMNs or M@SMNs at a final concentration of 200 µg mL^−1^ for 4 h. For CLSM, cells were washed with PBS, fixed with 4% paraformaldehyde for 10 min at room temperature, and stained with DAPI (1 µg mL^−1^) for 10 min to label nuclei. Samples were imaged using a CLSM system. For FCM, cells were harvested, washed with PBS, and resuspended in 500 µL PBS. Samples were analyzed using a MoFlo XDP flow cytometer, and data were processed with FlowJo software.

##### 4.2.8.3 Endocytic Pathway Analysis

RAW264.7 macrophages were seeded in 24‐well plates at a density of 2 × 10^5^ cells per well and incubated for 24 h to allow adherence. Cells were then pre‐treated with specific endocytosis inhibitors for 1 h to block different uptake pathways: 10 µg mL^−1^ chlorpromazine (clathrin‐mediated endocytosis), 50 µg mL^−1^ dynasore (dynamin‐dependent endocytosis), 2.5 mg mL^−1^ methyl‐β‐cyclodextrin (caveolae/lipid raft‐mediated endocytosis), 2.5 µg mL^−1^ cytochalasin D (actin‐dependent endocytosis), 50 µM amiloride (macropinocytosis), and 5 µM nocodazole (microtubule‐dependent endocytosis). Cells without inhibitor treatment served as controls. After pre‐treatment, the medium was replaced with fresh medium containing 100 µg mL^−1^ of nanoparticles, and cells were incubated for 4 h under standard culture conditions. Following incubation, cells were gently washed twice with PBS, detached in 500 µL PBS, and subjected to flow cytometry to quantify nanoparticle uptake.

#### 4.2.9 In Vivo Clearance Mechanisms of M@SMNs

##### 4.2.9.1 In Vivo and Ex Vivo Fluorescence Biodistribution Analysis

Cy5‐APTES was synthesized by dissolving 5 mg of Cy5‐SE in 5 mL of DMSO, followed by the addition of 26.3 µL of APTES (molar ratio of APTES:Cy5‐SE = 10:1). The reaction mixture was stirred at 300 rpm in the dark at room temperature for 24 h, obtaining the solution of Cy5‐APTES. The method for preparing Cy5‐labelled SMNs (Cy5‐SMNs) was similar to that for FITC‐labelled NPs.

BALB/c mice were intravenously administered Cy5‐labelled SMNs and Cy5‐labelled M@SMNs (Cy5‐M@SMNs) at a dose of 20 mg kg^−1^. In vivo fluorescence imaging was conducted using an in vivo imaging system (IVIS Lumina XRMS Series III, PerkinElmer, Inc., USA) 1 h post‐injection. Subsequently, the mice were euthanized and major organs (heart, liver, spleen, lungs, and kidneys) were collected for ex vivo imaging analysis.

##### 4.2.9.2 Quantification of FITC‐Labelled Nanoparticles in Serum

FITC‐labelled SMNs and M@SMNs were intravenously administered to mice at a dose of 20 mg kg^−1^. At 1 h post‐injection, mice were euthanized and blood samples were collected *via* the orbital sinus. Blood was allowed to stand at room temperature for 1 h, followed by centrifugation at 50 ×g for 10 min to isolate serum. A 100 µL aliquot of serum was diluted with 400 µL of ultrapure water, and the fluorescence intensity was measured using a multimode microplate reader (Spark, Tecan, Austria) to quantify circulating FITC‐labelled nanoparticles.

##### 4.2.9.3 Liver Cell‐Specific Uptake of SMNs and M@SMNs Ex Vivo

FITC‐labelled M@SMNs were intravenously administered to mice at a dose of 20 mg kg^−1^. 1 h post‐injection, the mice were anesthetized with isoflurane, and their thoracic cavities were opened. Preheated D‐Hank's solution (lacking Ca^2^^+^ and Mg^2^^+^ and containing 0.5 mM EDTA and 25 mM HEPES) was slowly perfused into the liver, followed by severing of the portal vein to enable the blood to be flushed from the liver. Once liver completely lost its red color, the perfusion solution was replaced by infusing with 10 mL of collagenase type IV to digest the liver tissue. The digested liver was collected, washed with PBS, and mechanically disrupted using forceps to release cells. The resulting cell suspension was filtered through a 100 µm mesh to obtain a liver cell suspension. Hepatocytes were pelleted by centrifugation at 50 ×g for 3 min, while the supernatant was centrifuged at 650 ×g for 10 min to isolate non‐parenchymal cells. The precipitate was resuspended in 3 mL of percoll solution and centrifuged at 400 ×g for 20 min. The upper layer containing cells was collected, mixed with 6 mL of PBS, and centrifuged again at 400 ×g for 5 min to isolate non‐parenchymal cells. To block Fc receptors, 1 µL of CD16/32 was added to 100 µL of non‐parenchymal cell suspension and incubated for 30 min. Subsequently, 1 µL of CD146 and 1 µL of F4/80 were added to label liver sinusoidal endothelial cells (LSECs) and Kupffer cells, respectively. FCM was performed to analyze the uptake of M@SMNs by different liver cells.

##### 4.2.9.4 Analysis of Nanoparticle Distribution Among Blood Immune Cell Populations

FITC‐labelled M@SMNs were administered to mice *via* tail vein injection at a dose of 20 mg kg^−1^. Blood samples were collected from the orbital sinus into anticoagulant tubes at 1 h post‐injection. To lyse erythrocytes, the three‐fold volume of erythrocyte lysis buffer was added to the collected blood, followed by incubation at 4°C for 10 min and centrifugation at 400 ×g for 10 min. The resulting pellet was resuspended in 2 mL of erythrocyte lysis buffer, lysed for an additional 10 min, and centrifuged at 400 ×g for 10 min to isolate the leukocyte pellet. A 100 µL aliquot of the leukocyte suspension was treated with 1 µL of CD16/32 for 30 min to block nonspecific binding. Subsequently, 2 µL of CD45, 1 µL of Ly6G, and 1.25 µL of CD11b were added to label neutrophils, while 2 µL of CD45, 1.25 µL of CD3, and 1.25 µL of CD19 were used to label T cells and B cells. FCM was then performed to analyze the uptake of M@SMNs by blood immune cells.

#### 4.2.10 Analysis of PC Formation and Its Impact on Cellular Uptake

##### 4.2.10.1 Preparation of Nanoparticle‐Protein Complexes

Mouse serum was mixed with an equal volume of SMNs or M@SMNs, followed by incubation with shaking at 300 rpm and 37°C for 1 h. To remove unbound serum proteins, the nanoparticle‐protein complexes were pelleted by centrifugation at 21,846 ×g and 4°C for 30 min. The supernatants were discarded, and the resulting pellets contained the nanoparticle‐protein complexes [91, 92].

##### 4.2.10.2 BCA Analysis

Protein adsorbed on nanoparticles was quantified using a BCA assay kit. Briefly, the bound proteins were first desorbed from nanoparticle surface by incubating the pellets with 100 µL of SDS‐Tris buffer (2 wt% SDS, 62.5 mM Tris‐HCl) at 95°C for 5 min. The detached proteins were separated from nanoparticles by centrifugation at 21,846 ×g and 4°C for 30 min. The supernatant was collected, and protein concentration was determined using the BCA assay kit according to the manufacturer's instructions.

##### 4.2.10.3 SDS‐PAGE Analysis

The serum proteins adsorbed on the nanoparticle surface were analyzed using SDS‐PAGE. The protein solutions recovered from the desorption step were mixed with protein loading buffer and then boiled at 100°C for 10 min. Subsequently, the samples were loaded onto a 4%–20% SDS‐PAGE gel. Gel electrophoresis was conducted at 150 V for 45 min, until the proteins with the lowest molecular weight reached the bottom of the gel. Protein bands were visualized by Coomassie blue staining.

##### 4.2.10.4 Cellular Uptake Analysis

RAW264.7 cells were seeded in 6‐well plates at a density of 5 × 10^5^ cells per well and cultured for 24 h for adherence. Cells were then incubated with either native M@SMNs or PC‐preincubated M@SMNs (100 µg mL^−^^1^) for 4 h. After incubation, cells were washed twice with PBS to remove unbound nanoparticles, harvested, and resuspended in 500 µL PBS. Cellular uptake was quantified by flow cytometry.

#### 4.2.11 Impact of PC Formation on Membrane Protein Accessibility of M@SMNs

##### 4.2.11.1 Preparation of Cy5‐Labelled Human Serum Proteins

Human serum proteins were labelled with Cy5 NHS ester (1 mg mL^−^^1^ in DMSO) via amine‐reactive conjugation. Specifically, 50 µL of Cy5 solution was added to 5 mL of human serum, gently vortexed, and incubated overnight at 4°C in the dark. Unreacted dye was removed by nine cycles of ultrafiltration using 3 kDa MWCO centrifugal filters (Millipore) at 7,140 ×g and 4°C for 30 min per cycle. The retained Cy5‐labelled proteins were resuspended in PBS, quantified by BCA assay, adjusted to 20 mg mL^−^^1^, and stored at 4°C protected from light until use.

##### 4.2.11.2 Single‐Particle Quantification of Serum Protein Adsorption

FITC‐labelled SMNs or M@SMNs (500 µL, 1 mg mL^−^^1^) were incubated with Cy5‐labelled human serum proteins (500 µL, 20 mg mL^−^^1^; v/v = 1:1) at 37°C for 1 h to allow protein corona (PC) formation. Following incubation, the samples were centrifuged at 21,846 ×g and 4°C for 30 min to remove the supernatant and then washed three times with PBS to eliminate unbound proteins. NanoFCM was subsequently employed to simultaneously detect FITC signals from nanoparticles and Cy5 signals from adsorbed serum proteins at the single‐particle level.

##### 4.2.11.3 Single‐Particle Quantification of Serum Protein Masking on CD86 and CD206

M1@SMNs and M2@SMNs (500 µL, 1 mg mL^−^^1^) were incubated with or without human serum (v/v = 1:1, 37°C, 1 h), followed by centrifugation at 21,846 ×g and 4°C for 30 min to remove the supernatant and then washed 3 times with PBS to eliminate unbound proteins. After washing, M1@SMNs were stained with APC‐anti‐CD86 antibody (2 µL, 100 µg/mL), and M2@SMNs with APC‐anti‐CD206 antibody (2 µL, 100 µg/mL), at room temperature in the dark for 1 h. Unbound antibodies were removed by centrifugation at 21,846 ×g for 30 min. The nanoparticles resuspended in PBS, and analyzed by NanoFCM.

##### 4.2.11.4 NanoFCM Instrument Calibration

NanoFCM was calibrated using monodisperse silica nanoparticles of 50, 100, and 200 nm diameters for side scatter (SSC) intensity scaling. The fluorescence detection channels (FITC and Cy5) were optimized using control particles labelled with corresponding fluorophores. For each sample, at least 10,000 events were collected to ensure statistical reliability.

#### 4.2.12 Liquid Chromatography‐Tandem Mass Spectrometry (LC‐MS/MS) Analysis

Freshly prepared nanoparticle–protein complexes formed by incubation with mouse serum were resuspended in 2% SDS solution and heated at 95°C for 10 min to desorb adsorbed proteins. After centrifugation at 21,846 ×g for 30 min, the supernatants were collected for protein quantification using a BCA assay kit. For protein reduction and alkylation, dithiothreitol (DTT) was added to a final concentration of 5 mM and incubated at 56°C for 25 min, followed by alkylation with 14 mM iodoacetamide (IAM) in the dark at room temperature for 30 min. Proteins were then digested with trypsin at a mass ratio of 1:100 (trypsin:protein) at 37°C for 18 h. The digestion was quenched with 100 µL of 99% acetic acid. Peptides were desalted, vacuum lyophilized, and reconstituted in 0.1% (v/v) formic acid. Subsequently, the peptides were separated using an EASY‐nLC 1200 system (Thermo Fisher Scientific, USA) and analyzed on a Q Exactive HF‐X mass spectrometer (Thermo Fisher Scientific, USA). Tandem mass spectrometry (MS/MS) data were searched against the mouse UniProt protein database using Proteome Discoverer software (Thermo Fisher Scientific).

#### 4.2.13 Evaluation of Complement Activation using Enzyme‐Linked Immunosorbent Assay

##### 4.2.13.1 ELISA Analysis

Complement activation was assessed by quantifying the serum concentrations of C3a and C5a using commercial ELISA kits, following the manufacturer's instructions. Nanoparticles were incubated with mouse or human serum at a 1:1 volume ratio (v/v) at 37°C for 1 h. The reaction was terminated by adding PBS containing 40 mM EDTA. After centrifugation at 21,846 ×g for 30 min, the supernatant was collected, and the levels of C3a and C5a were determined using species‐specific ELISA kits. Zymosan (3 mg mL^−^^1^) and PBS were used as positive and negative controls, respectively. All experiments were performed in triplicate.

##### 4.2.13.2 WB Analysis

M@SMNs were incubated with mouse serum at a 1:1 volume ratio at 37°C and 300 rpm for 1 h. After incubation, samples were centrifuged at 21,846 ×g for 30 min at 4°C to collect the nanoparticle–protein complexes. The pellet was resuspended in 100 µL of 2% SDS solution and heated at 95°C for 10 min to desorb bound proteins. Following a second centrifugation (21,846 ×g, 30 min), the supernatant containing the eluted proteins was collected and quantified using a BCA protein assay kit. Equal amounts of protein (10 µg per lane) were separated on 8% SDS‐PAGE gels and transferred onto nitrocellulose (NC) membranes at 300 mA for 90 min. Membranes were washed with TBST (3 × 5 min), blocked overnight in a rapid blocking buffer, and then incubated with primary anti‐C3 antibody (dilution as recommended) at 4°C for 12 h. After washing (3 × 5 min with TBST), membranes were incubated with HRP‐conjugated secondary antibody for 1 h at room temperature. After additional washes (5 × with TBST), the membranes were developed using chemiluminescent substrate and imaged using a gel documentation system.

#### 4.2.14 Statistical Analysis

Statistical analysis was performed using GraphPad Prism software (Version 8.0.2). Data were obtained from at least three independent experiments, with all quantitative parameters presented as mean ± standard deviation (SD). For comparisons between two groups, Student's t‐test was used. For multiple group comparisons, one‐way analysis of variance (ANOVA) was applied. Statistical significance was defined as follows: *p < 0.05, **p < 0.01, ***p < 0.001, ****p < 0.0001, with n.s. indicating no significant difference.

## Author Contributions

Supervision: K.L., and S.J. Conceptualization: T.C.H., L.N.Z., V.M., D.C., K.L., and S.J. Methodology: T.C.H., and L.N.Z. Investigation: T.C.H., L.N.Z., J.Y.D., X.Y.F., and Y.G. Visualization: T.C.H., L.N.Z., and S.J. Writing (original draft): T.C.H., and L.N.Z. Writing (review and editing): V.M., D.C., K.L., and S.J. All authors discussed the results and commented on the paper.

## Funding

This work was financially supported by National Science Foundation of China (32201149) and Shandong Provincial Natural Science Fund for Excellent Young Scientists Fund Program (Overseas) (2022HWYQ‐063). D.C. and S.J. acknowledge the support of the grant “Re‐inventing University” of the Ministry of Higher Education, Science, Research and Innovation (MHESI).

## Conflicts of Interest

The authors declare no conflict of interest.

## Supporting information




**Supporting File**: smll72877‐sup‐0001‐SuppMat.docx.

## Data Availability

The data that support the findings of this study are available from the corresponding author upon reasonable request.
