# Supplementary material for: Macrophage Phenotype–Dependent Protein Corona Formation Governs Ligand Accessibility and Immune Clearance of Biomimetic Nanoparticles
Source: Small. 2026 Feb 20;22(22):e14389. doi: 10.1002/smll.202514389 (PMC13089100; doi:10.1002/smll.202514389)
Supplement: Supplementary file 1 — Supporting File: smll72877‐sup‐0001‐SuppMat.docx. [file SMLL-22-e14389-s001.docx]

**Supporting Information**

**Macrophage Phenotype–Dependent Protein Corona Formation Governs Ligand Accessibility and Immune Clearance of Biomimetic Nanoparticles**

*Tianchang He^#^, Lina Zhu^#^, Jiayi Ding, Xiaoyan Fang, Yu Gao, Volker Mailänder, Daniel Crespy, Katharina Landfester*, Shuai Jiang**

T. He, L. Zhu, J. Ding, X. fang, Y. Gao, S. Jiang

Key Laboratory of Marine Drugs, Chinese Ministry of Education, School of Medicine and Pharmacy, Ocean University of China, Qingdao 266003, P. R. China

State Key Laboratory of Marine Food Processing and Safety Control, Ocean University of China, Qingdao 266404, P. R. China

Laboratory for Marine Drugs and Bioproducts, Qingdao Marine Science and Technology Center, Qingdao 266237, China

V. Mailänder

Max Planck Institute for Polymer Research, Ackermannweg 10, 55128 Mainz, Germany

Dermatology Department, University Medicine Mainz, Langenbeckstr. 1, 55131 Mainz, Germany

D. Crespy

Department of Materials Science and Engineering, School of Molecular Science and Engineering, Vidyasirimedhi Institute of Science and Technology (VISTEC), Rayong 21210, Thailand

K. Landfester

Max Planck Institute for Polymer Research, Ackermannweg 10, 55128 Mainz, Germany

*Shuai Jiang, Email: jiangshuai@ouc.edu.cn

Katharina Landfester, Email: [landfester@mpip-mainz.mpg.de](mailto:landfester@mpip-mainz.mpg.de)

^#^These authors contributed equally to this work

**Figure S1.** Particle size distribution of SMNs in water measured using dynamic light scattering (DLS).


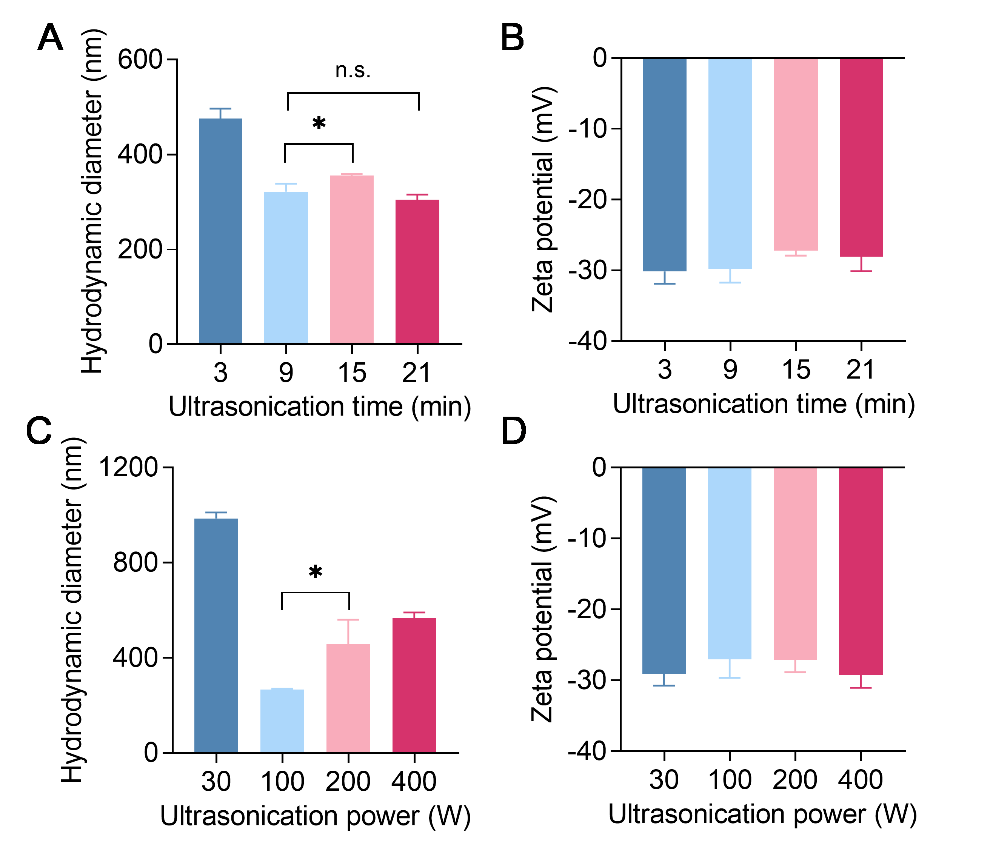


**Figure S2. Optimization of the preparation process for macrophage membrane-coated nanoparticles (M@SMNs).** (A) Hydrodynamic diameter (*D*_h_) and (B) zeta potential of M@SMNs prepared with different ultrasonication durations. Data are presented as mean ± SD (*n* = 3) and statistically analyzed using student’s *t* test. *P < 0.05, n.s., not significant. (C) *D*_h_ and (D) zeta potential of M@SMNs prepared with varying ultrasonication powers. Data are presented as mean ± SD (*n* = 3) and statistically analyzed using student’s *t* test. *P < 0.05.

**Figure S3.** Polydispersity index (PDI) of SMNs and M@SMNs coated with different macrophage phenotypes (M0, M1, and M2), as measured by DLS in water. Data are presented as mean ± SD (*n* = 3).

**Figure S4.** Zeta potentials of M@SMNs and CVs. Data are presented as mean ± SD (*n* = 3).


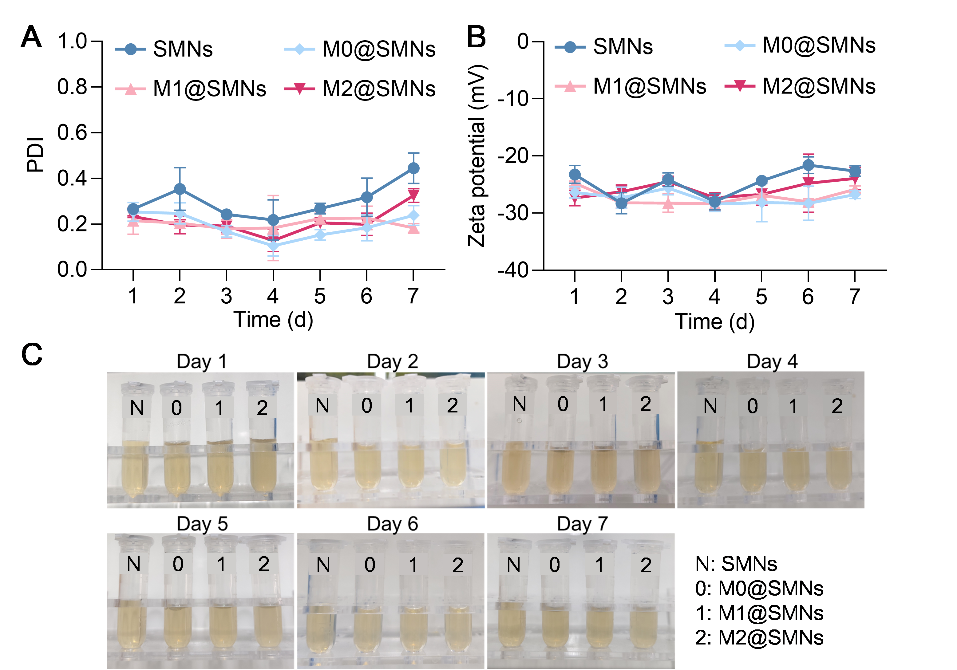


**Figure S5. Stability assessment of SMNs and M@SMNs derived from different phenotypes.** (A) PDI. (B) Zeta potential. Data are presented as mean ± SD (*n* = 3). (C) Visual appearance from photographs.


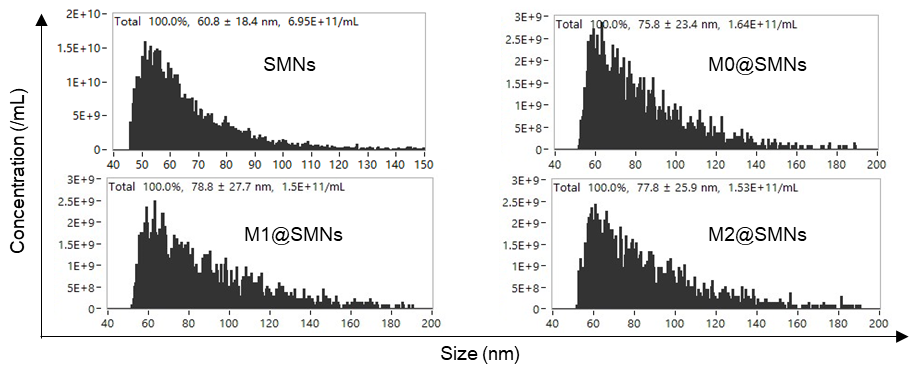


**Figure S6.** Size distribution profiles of M@SMNs obtained by NanoFCM analysis.


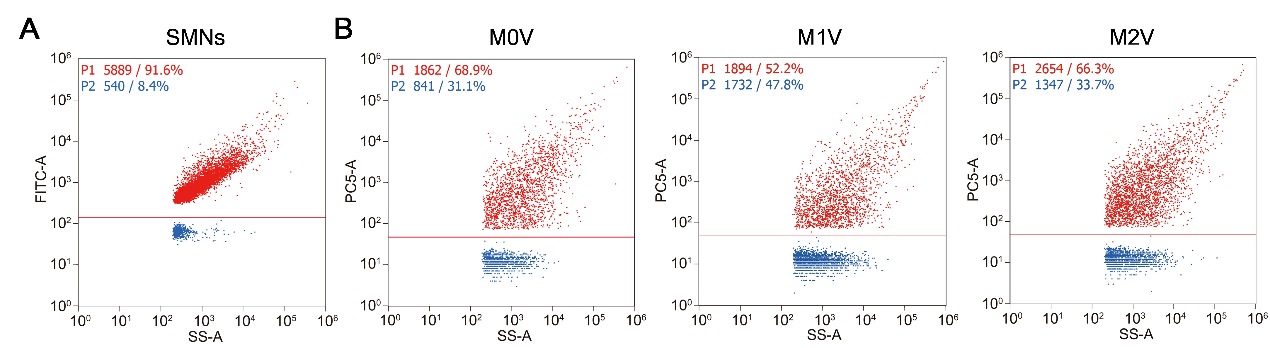


**Figure S7. Fluorescence labelling rates of SMNs and cell membrane vesicles detected by NanoFCM.** (A) FITC-labelling rate of SMNs. (B) DiD-labelling rate of cell membrane vesicles.

**Figure S8.** Fluorescence spectra of M@SMNs after incubation with 1,8-ANS probe analyzed using the fluorescence spectrometer.


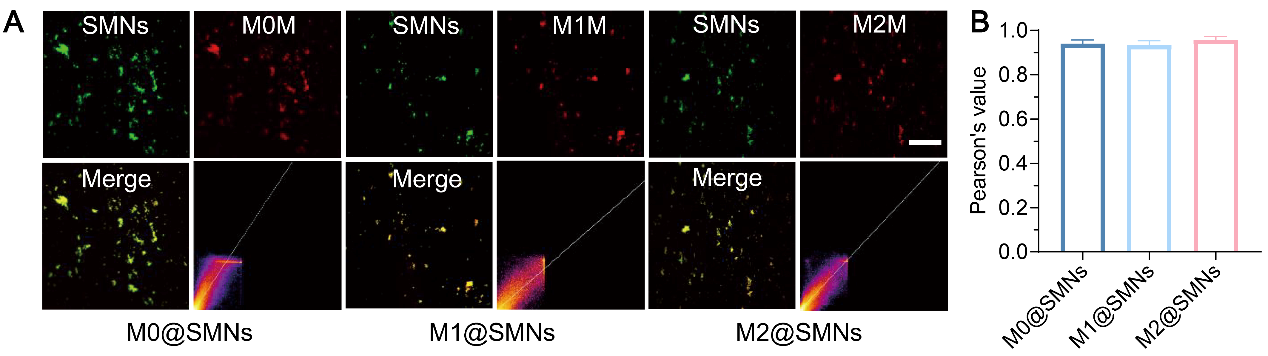


**Figure S9. Fluorescence colocalization characterization.** (A) Confocal laser scanning microscopy (CLSM) images showing SMNs labelled with FITC (green) and macrophage membranes (M0M/M1M/M2M) labelled with DiD (red). Scale bar: 20 *μ*m. (B) Pearson’s correlation coefficient for colocalization, calculated using ImageJ software. Data are presented as mean ± SD (*n* = 3).

**
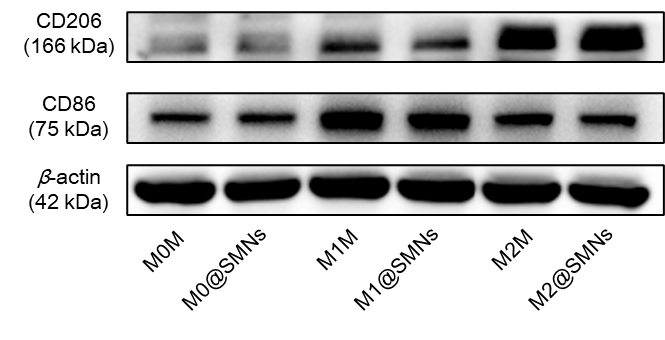
**

**Figure S10.** Membrane protein bands from macrophage membranes and M@SMNs analyzed using WB.

**
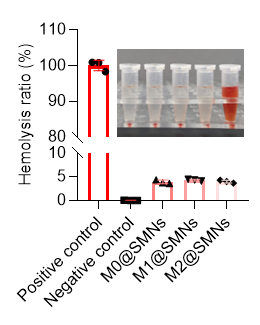
**

**Figure S11.** Hemocompatibility of SMNs and M@SMNs evaluated on rat red blood cells, with PBS and water serving as negative and positive controls, respectively. Data are presented as mean ± SD (*n* = 3).

**Figure S12.** Quantitative analysis of fluorescence intensities of the liver upon treatment with SMNs and M@SMNs. Data are presented as mean ± SD (*n* = 3) and statistically analyzed using student’s *t* test. *P < 0.05, **P < 0.01, n.s., not significant.


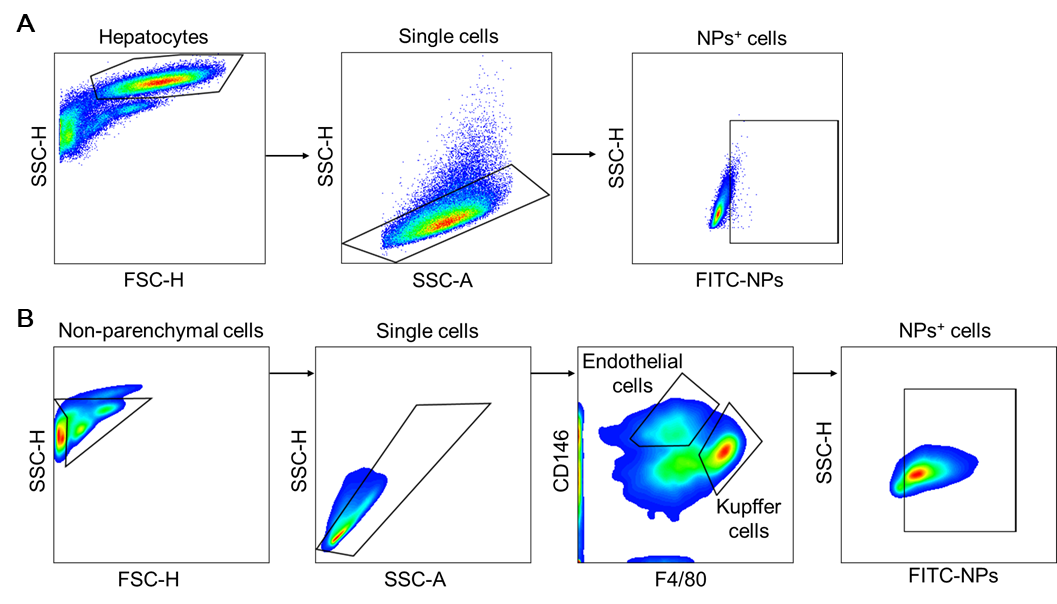


**Figure S13. Gating strategy for FCM analysis of M@SMN uptake by liver cells.** (A) Hepatocytes. (B) Non-parenchymal cells.

**
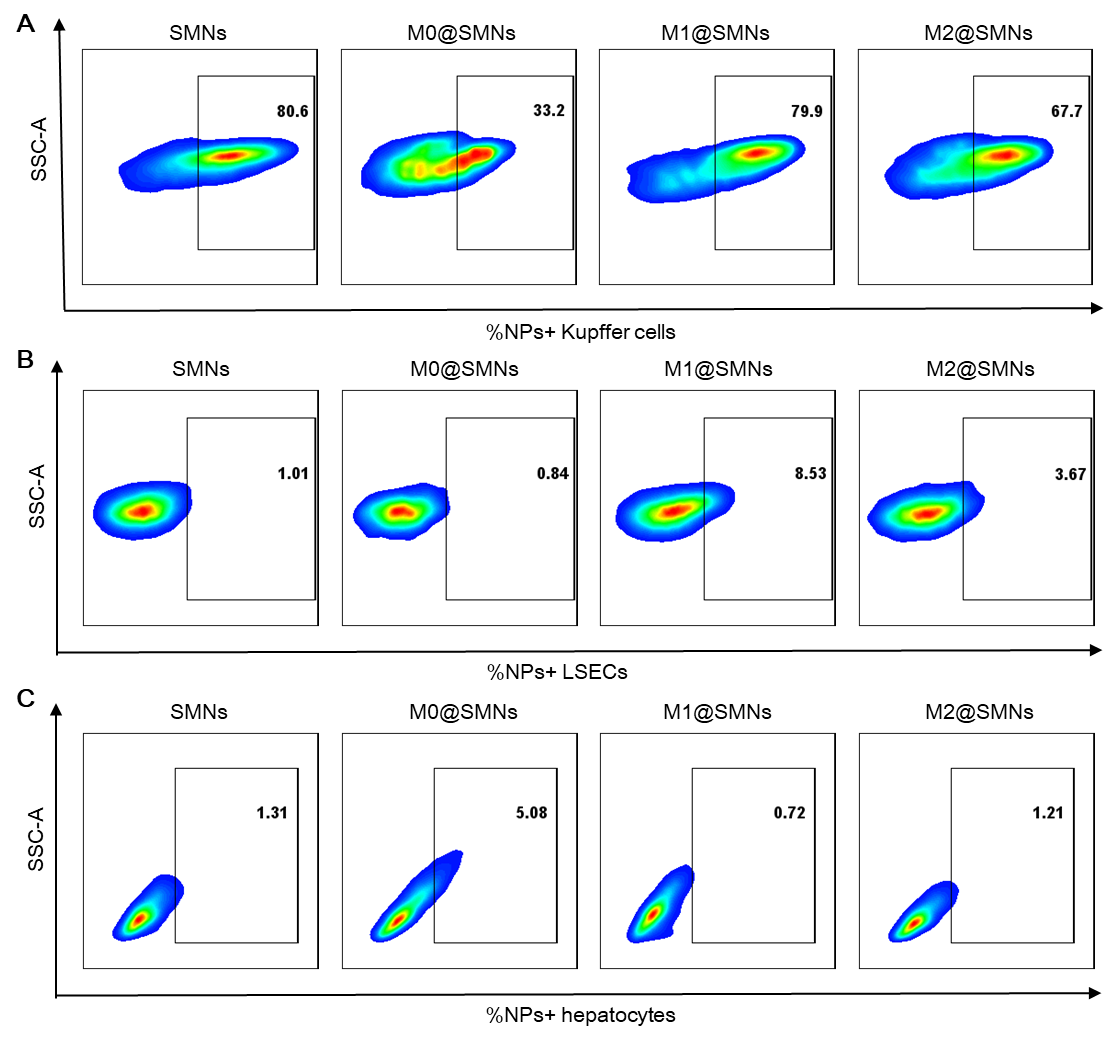
**

**Figure S14. FCM scatter plot illustrating nanoparticle uptake by liver cells.** (A) Kupffer cells. (B) LSECs. (C) Hepatocytes.

**
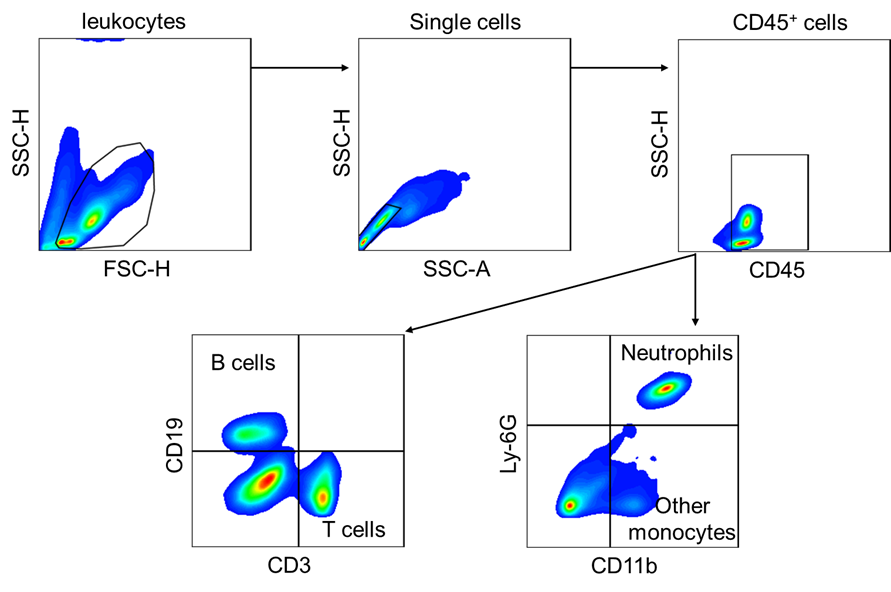
**

**Figure S15.** Gating strategy for FCM analysis of M@SMN uptake by blood immune cells.

**
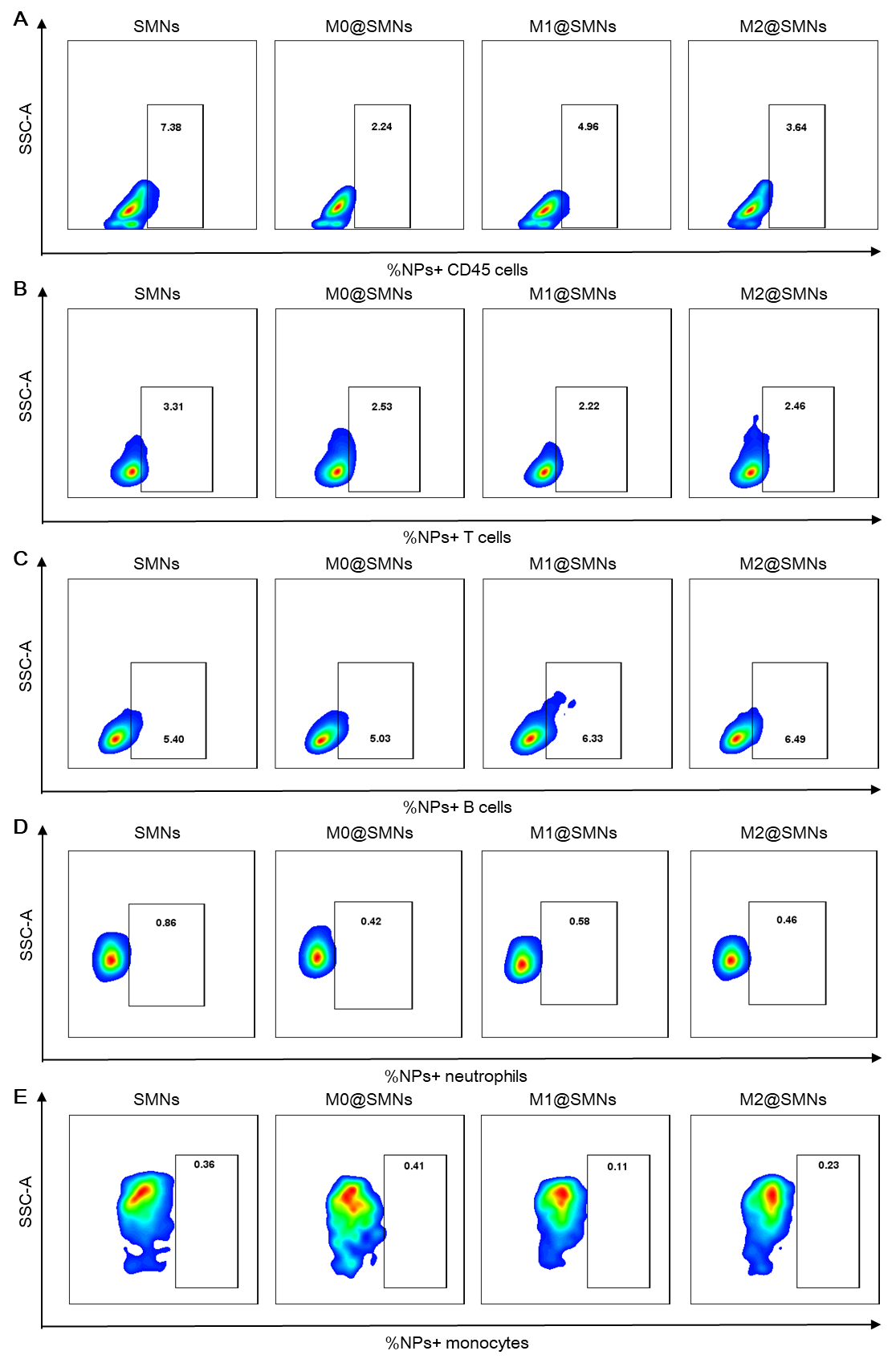
**

**Figure S16.** **FCM scatter plots showing the uptake of nanoparticles by various blood immune cell populations.** (A) CD45 cells. (B) T cells. (C) B cells. (D) Neutrophils. (E) Monocytes.


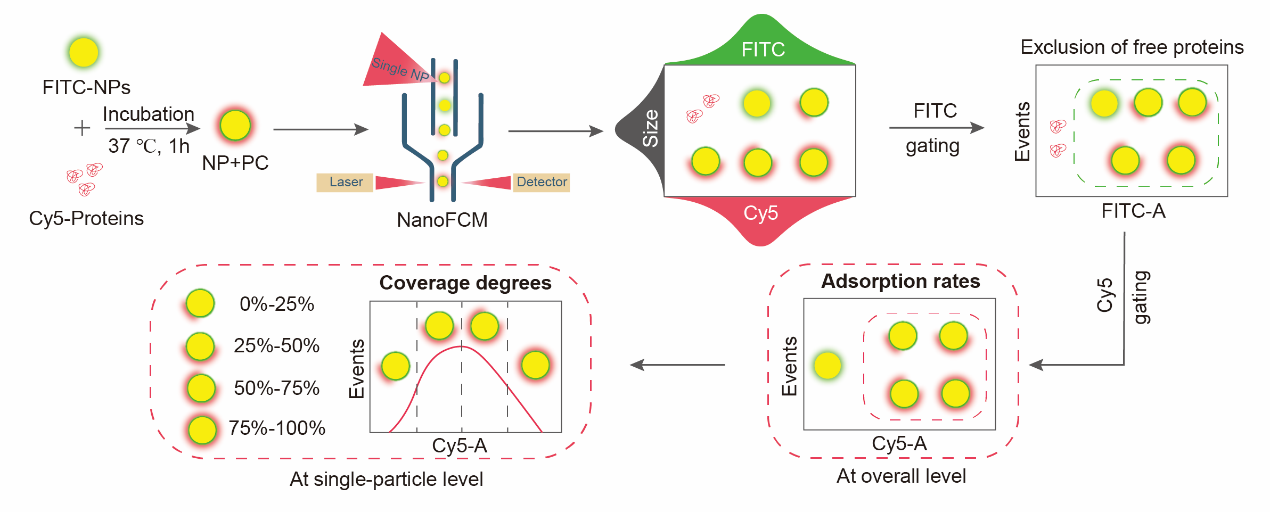


**Figure S17.** Schematic illustration of single-particle analysis for monitoring PC formation on nanoparticles using NanoFCM.


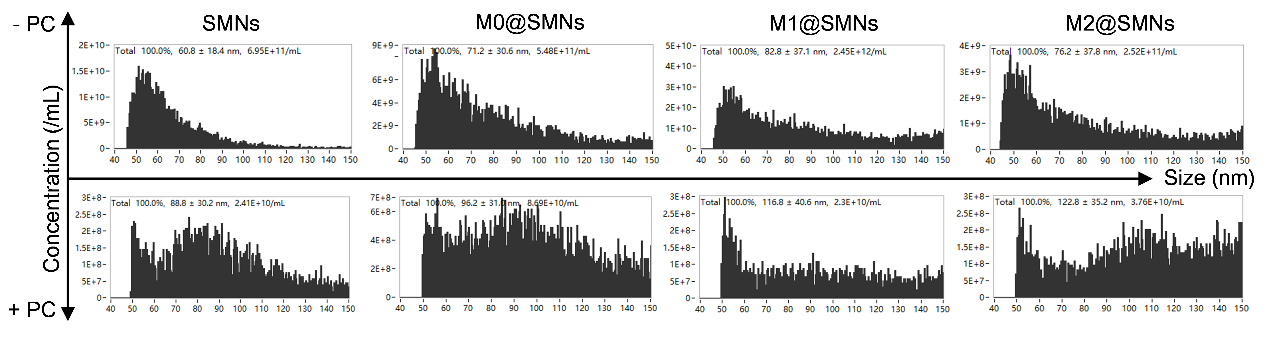


**Figure S18.** Changes in particle size distribution of M@SMNs before and after serum incubation, as measured by NanoFCM.


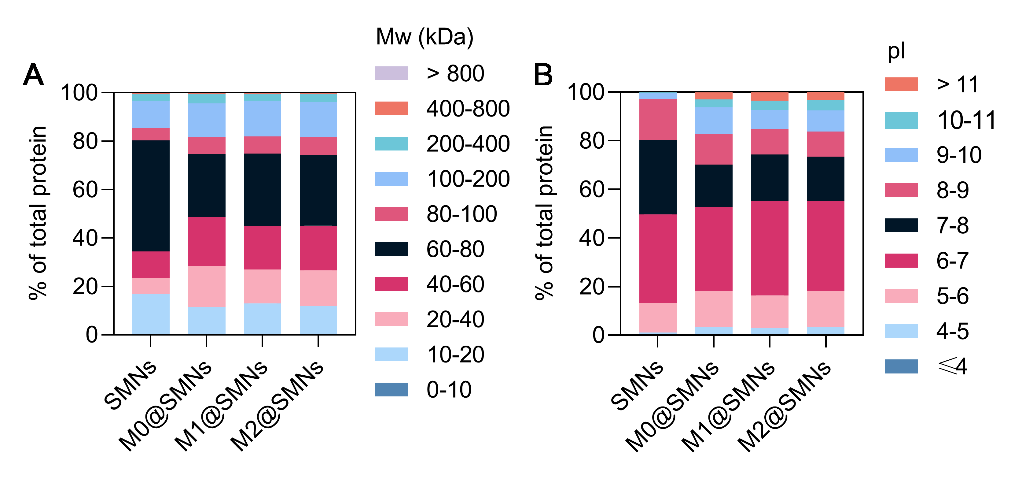


**Figure S19.** Classification of corona proteins according to (A) molecular weight (*M*_w_) and (B) isoelectric point (pI).


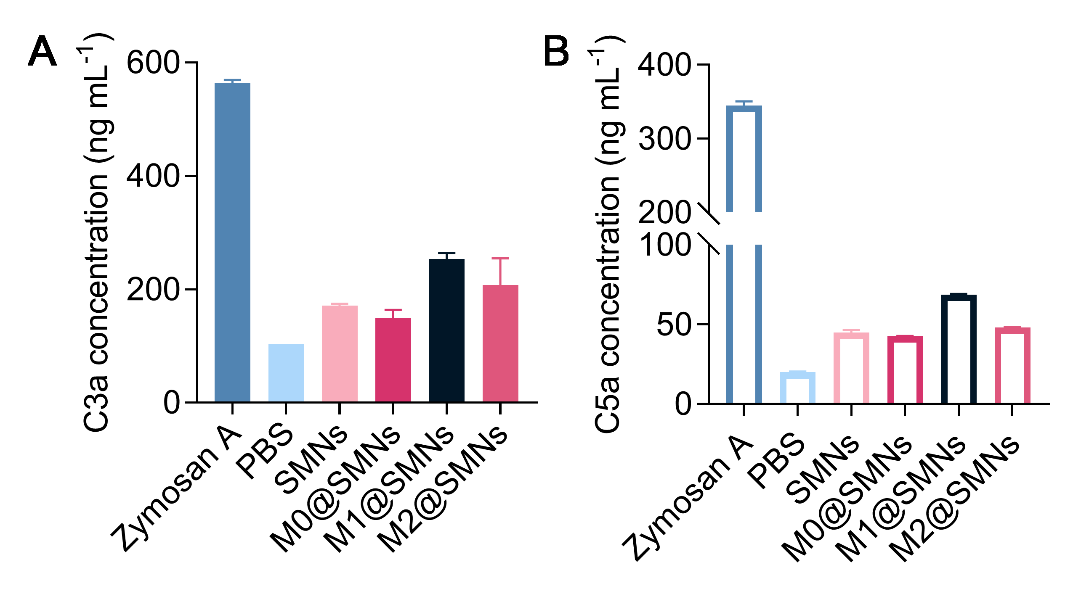


**Figure S20.** ELISA analysis of complement activation products (A) C3a and (B) C5a in mouse serum induced by SMNs and M@SMNs, with PBS and Zymosan (3 mg mL^-1^) as negative and positive controls, respectively. Data are presented as mean ± SD (*n* = 3).
